# Isolating Discriminant Neural Activity in the Presence of Eye Movements and Concurrent Task Demands

**DOI:** 10.3389/fnhum.2017.00357

**Published:** 2017-07-07

**Authors:** Jon Touryan, Vernon J. Lawhern, Patrick M. Connolly, Nima Bigdely-Shamlo, Anthony J. Ries

**Affiliations:** ^1^U.S. Army Research Laboratory, Future Soldier Technologies Division, Human Research and Engineering Directorate, Aberdeen Proving GroundAberdeen, MD, United States; ^2^Teledyne Scientific CompanyDurham, NC, United States; ^3^Qusp LabsSan Diego, CA, United States

**Keywords:** fixation-related potentials, EEG, eye tracking, target detection, cognitive load

## Abstract

A growing number of studies use the combination of eye-tracking and electroencephalographic (EEG) measures to explore the neural processes that underlie visual perception. In these studies, fixation-related potentials (FRPs) are commonly used to quantify early and late stages of visual processing that follow the onset of each fixation. However, FRPs reflect a mixture of bottom-up (sensory-driven) and top-down (goal-directed) processes, in addition to eye movement artifacts and unrelated neural activity. At present there is little consensus on how to separate this evoked response into its constituent elements. In this study we sought to isolate the neural sources of target detection in the presence of eye movements and over a range of concurrent task demands. Here, participants were asked to identify visual targets (Ts) amongst a grid of distractor stimuli (Ls), while simultaneously performing an auditory N-back task. To identify the discriminant activity, we used independent components analysis (ICA) for the separation of EEG into neural and non-neural sources. We then further separated the neural sources, using a modified measure-projection approach, into six regions of interest (ROIs): occipital, fusiform, temporal, parietal, cingulate, and frontal cortices. Using activity from these ROIs, we identified target from non-target fixations in all participants at a level similar to other state-of-the-art classification techniques. Importantly, we isolated the time course and spectral features of this discriminant activity in each ROI. In addition, we were able to quantify the effect of cognitive load on both fixation-locked potential and classification performance across regions. Together, our results show the utility of a measure-projection approach for separating task-relevant neural activity into meaningful ROIs within more complex contexts that include eye movements.

## Introduction

Goal-directed eye movements are a ubiquitous component of everyday life and integral to our perception of the world. Over recent decades, numerous visual search studies have used eye movement patterns to better understand perceptual and attentional processes that underlie human vision (Kowler, 2011). In contrast, the majority of human electrophysiological studies of visual search continue to use fixation constrained paradigms, artificially limiting the natural linkage between attentional shifts and subsequent eye movements. Thus, extending these paradigms into a framework of overt visual search would enable the validation of attentional models in a more natural context. However, a number of potential confounds and analytical challenges emerge when interpreting electroencephalography (EEG) in the presences of eye movements (Nikolaev et al., 2016). One of the primary confounds is the large eye movement related signals around the events of interest, namely saccades and fixations. These include the corneo-retinal and saccadic spike potentials along with eyelid artifacts. Importantly, the magnitude of these signals systematically scale with the direction, amplitude, and velocity of the saccade. Furthermore, saccade features themselves can systematically vary with task design or conditions. To address this concern, a number of methods have been developed to account for the effects of saccade sequence (Dandekar et al., 2012b) or isolate eye movement related signals within the EEG record (Plöchl et al., 2012). These approaches have been successful to the degree that they were able to reveal task-relevant activity, such as the P3 component, that may otherwise have been conflated with eye movement related artifacts (Dandekar et al., 2012a; Devillez et al., 2015).

Robust saccade detection and quantification presents another methodological challenge. Previously, EEG studies often relied on an explicit electrooculography (EOG) measurement, horizontal or vertical, for detecting the onset of a saccade (Gaarder et al., 1964; Thickbroom et al., 1991; Kazai and Yagi, 1999). The benefit of using this signal is both the high temporal resolution and the *de facto* alignment with the EEG record. Unfortunately, the lack of precision in determining the direction and distance of saccades limits these studies to paradigms with a small number of predetermined fixation locations. However, recent advances in the speed and accuracy of infrared eye-tracking technology has made it possible to link gaze position with neural activity at both high spatial and temporal resolution. This has led to a growing number of studies that explore the neural correlates of target detection during visual search, in both controlled (Brouwer et al., 2013) and free-viewing paradigms (Kamienkowski et al., 2012; Dias et al., 2013; Jangraw et al., 2014; Kaunitz et al., 2014; Ušćumlić and Blankertz, 2016; Wenzel et al., 2016).

In addition to the above measurement and signal processing challenges, there is the more nuanced task of interpreting brain activity in the context of planned and executed eye movements (Nikolaev et al., 2016). This remains a significant obstacle for studies focusing on both perceptual and cognitive phenomena. First, there is the task of quantifying or controlling for stimulus properties. When the eyes are free to move, stimuli impinging on the retina will necessarily vary across conditions and participants, even when gaze position is guided by the task sequence. In more controlled settings, experimental design can ensure that small differences in eye position do not significantly bias the statistics of the stimuli. However, this becomes more challenging for the ultimate goal of free-viewing in natural scenes where spatial frequency, orientation, and chromatic distributions can vary widely within a single image. Likewise, there is the challenge of separating saccade planning and execution from the perceptual or cognitive signal of interest. Even when utilizing high density EEG and source localization techniques, the spatial resolution of the saccadic preparatory signals is limited. Thus, accounting for these signals via subtraction across equated conditions (Nikolaev et al., 2013), regression (Dandekar et al., 2012b), or other techniques (Dias et al., 2013) is an important factor for the interpretation of para-saccadic neural activity.

Despite these recent advancements, there remains a need for development and validation of methods for the quantification of both perceptual and cognitive phenomena in the presence of eye movements. Part of this process is the evaluation of novel analytical approaches within paradigms that enable a more direct comparison to related fixation-constrained studies. Similarly, any particular method may only address some of the above challenges while still providing valuable insight when applied within the appropriate constraints or combined with other techniques. It is within this context that we propose the following approach for separating neural activity into meaningful regions of interest (ROIs) in the presence of eye movements. To evaluate our approach, we utilized data from a previously publish study (Ries et al., 2016) that employed a dual-task paradigm, *visual target detection* and *auditory N-back*, to quantify the effect of working memory load on the lambda response. The primary observation from this study was a small but significant reduction in the lambda amplitude with increasing cognitive load.

Here, we were able to separate the neural response to each fixation into six ROIs by applying a technique that linearly combines activity from independent sources based on their equivalent dipole location. Within each ROI we show a distinct neural response that, to varying degrees, discriminated target from non-target fixations and was differentially modulated by cognitive load. While the task design mitigated the overlapping response from adjacent saccades, common in free-viewing visual search, this approach is a substantive step in the interpretation of fixation-related brain activity. When combined with GLM-based techniques for the deconvolution of overlapping FRPs, this approach can be applied to more natural contexts where the interplay between bottom-up and top-down neural activity is not well understood.

## Materials and methods

The experiment used in this study has been described in a previous publication (Ries et al., 2016). Here, we provide a summary of stimuli and procedure, followed by a more detailed description of the novel ROI analysis method.

### Participants

Fourteen participants volunteered for the study; all participants were right-handed males with an average age of 32.8 years. All participants had 20/20 vision or corrected to 20/20 vision. This study was conducted in accordance with the U.S. Army Research Laboratory's IRB requirements (32 CFR 219 and DoDI 3216.02). The voluntary, fully informed consent of research participants was obtained in written form. The study was reviewed and approved by the U.S. Army Research Laboratory's IRB before the study began.

### Stimuli and procedure

**Figure 1:**
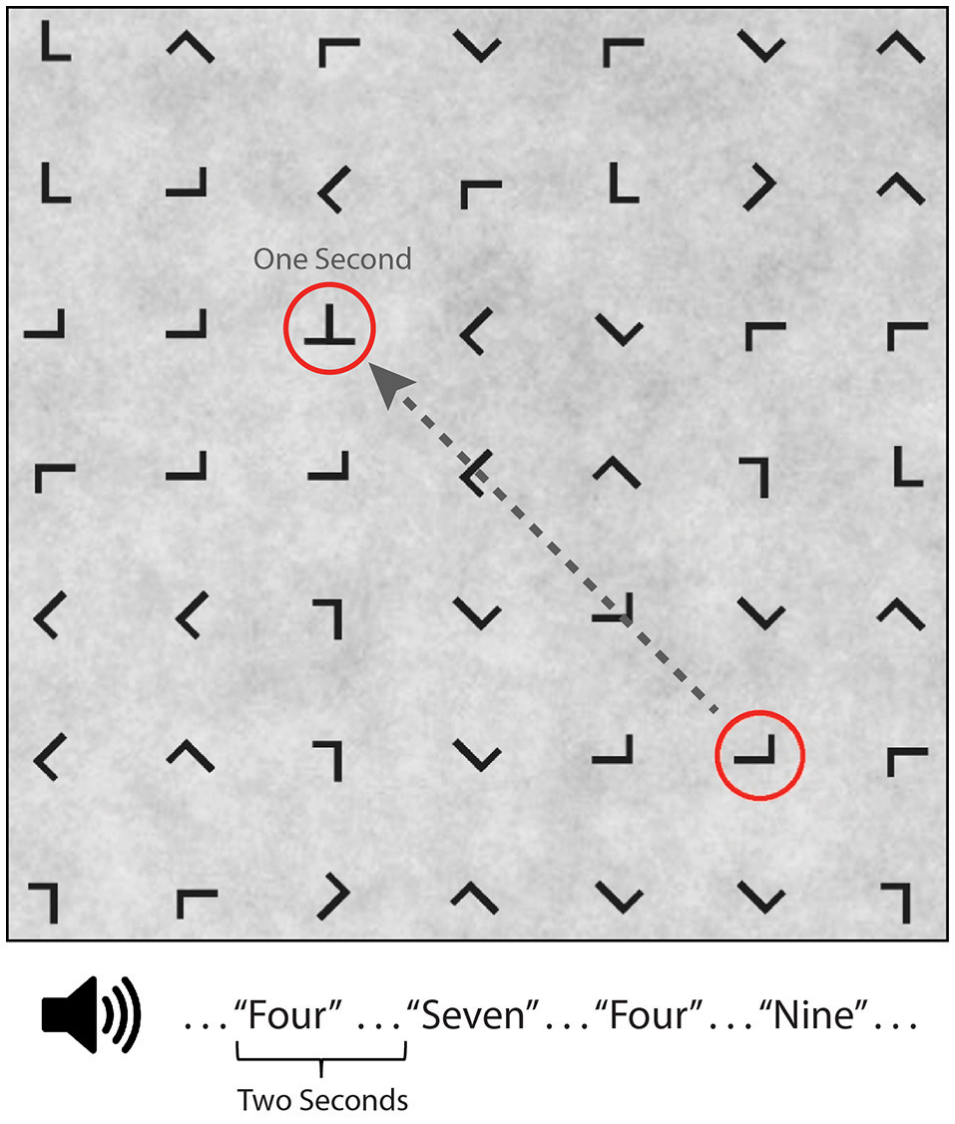
Guided visual target detection task with concurrent auditory N-back task. Example search grid containing target (“T”) and non-target (“L”) stimuli. A red annuls, surrounding one character, randomly shifted grid location every second. Auditory stimuli consisted of digits (numbers 0–9), binaurally presented 500 ms after annuls onset. Auditory task conditions included silent (no auditory stimuli), ignore, 0-back, 1-back, and 2-back.

Participants performed a guided visual target detection task on a 7 × 7 grid (23.9° × 23.9° visual angle) of equally spaced and variably oriented “T” or “L” characters (1.1° visual angle) presented on a low contrast 1/f noise background from a viewing distance of approximately 65 cm (Figure 1). Eye fixations were guided across the grid by a red annulus (2.3° visual angle) that randomly surrounded one of the characters for a duration of 1 s before moving to the next randomly selected character. Participants were instructed to saccade to and fixate on the character in the center of the red annulus and to press a button (left hand) only when a “T” (visual target) was present. Visual target characters appeared on 10% of trials. Participants were instructed to maintain fixation on the character until the next red annulus appeared. All red annuli surrounding a non-target “L” were at least two characters from any “T” present on the grid to minimize peripheral detection. The guided visual target detection task was performed in one of five conditions: visual alone (silent condition), while ignoring binaurally presented digits (numbers 0–9), or while using the auditory digits in a 0, 1, or 2-back working memory task. The digit “0” was only used in the 0-Back condition where it served as the auditory target. Auditory stimuli were presented every 2 s with a 500 ms offset from a shift in the red annulus location. Participants were instructed to make a button press (right hand) for auditory targets, which occurred on 20% of trials. Thus, the same number of targets appeared in both tasks during the 0-back, 1-back, and 2-back conditions. Participants performed two consecutive blocks of the same condition (silent, ignore, 0-back, 1-back, 2-back) with the condition order counterbalanced. Each of the 10 blocks had a duration of 200 s with self-paced rest periods between blocks. Participants were given practice in each N-back condition, prior to experimental data collection, until they reached above chance performance.

### Eye tracking

**Figure 2:**
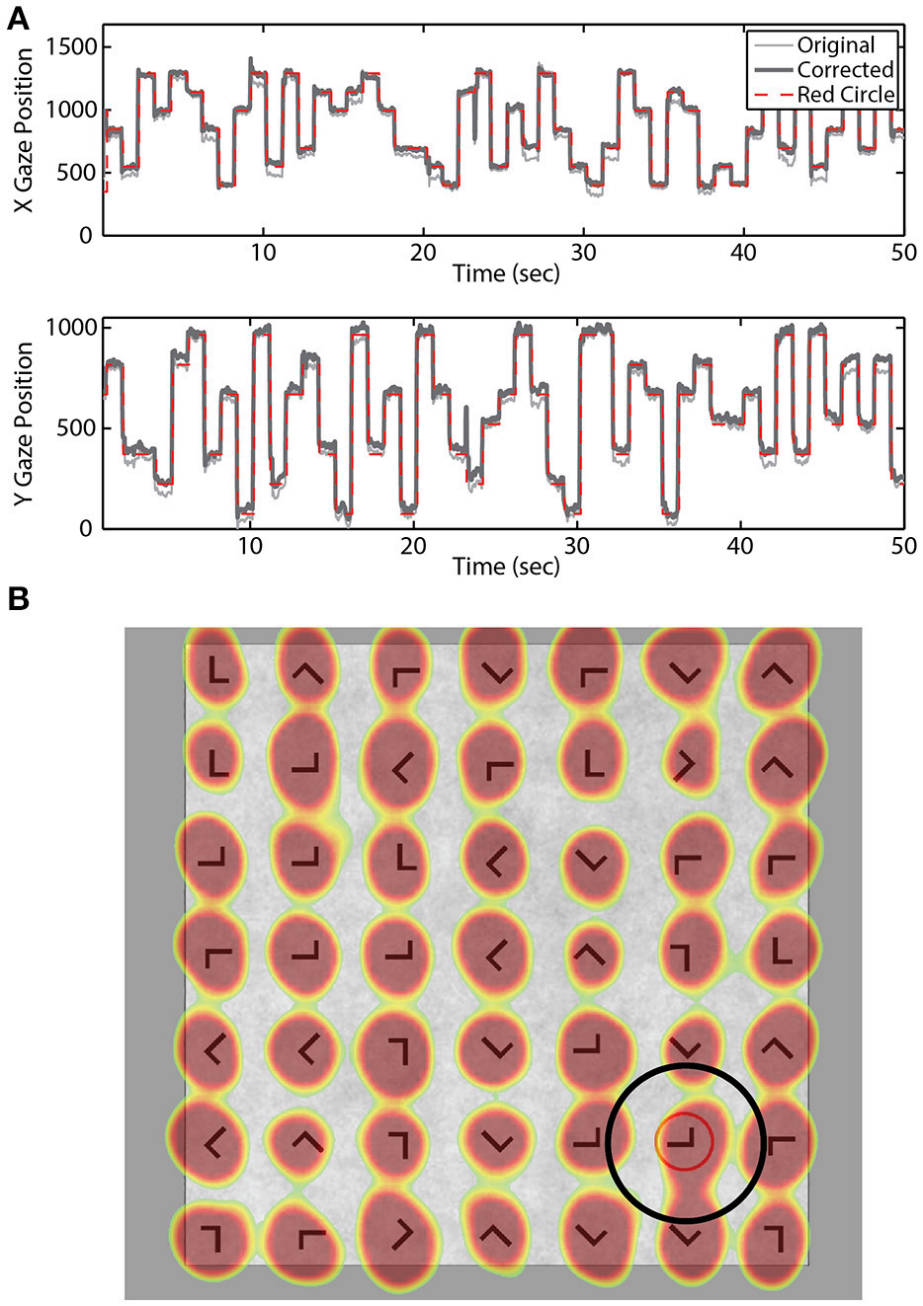
Gaze location estimation. **(A)** Example X and Y gaze vectors before and after correction. Dashed red line indicates location of the red annulus. **(B)** Example search grid overlaid with aggregate gaze position across all blocks for one participant. Black circle illustrates the fixation area considered valid for that stimulus location.

Eye-tracking data were sampled at 250 Hz using the SMI RED 250 system (Teltow, Germany). A 15-point calibration was performed prior to the practice and experimental blocks. A *post-hoc* model was fit to the eye-tracking data for each participant to increase accuracy of the gaze position estimate. Briefly, we used the expected eye position (i.e., location of the red annulus) to fit a quadratic regression model for both the horizontal and vertical gaze position vectors (Figure 2). A temporal lag (250 ms) was applied to the expected location (red annulus) to account for the delay between annulus onset and subsequent fixation.

Saccades and fixations were detected in the eye-tracking data using a velocity-based algorithm (Engbert and Mergenthaler, 2006; Dimigen et al., 2011). Saccades and fixations were detected using a velocity factor of 6 (standard deviations of the velocity distributions), minimum saccade duration of 20 ms, minimum fixation duration of 350 ms. If two saccades occurred within a 350 ms window, only the fixation corresponding to the largest saccade was preserved. Fixations were only considered task-relevant or “valid” if they were within 3 degrees of the current stimulus location. These criteria were chosen to focus analyses on the first saccade onto the new stimulus (red annulus) location.

### Electroencephalography and feature extraction

**Figure 3:**
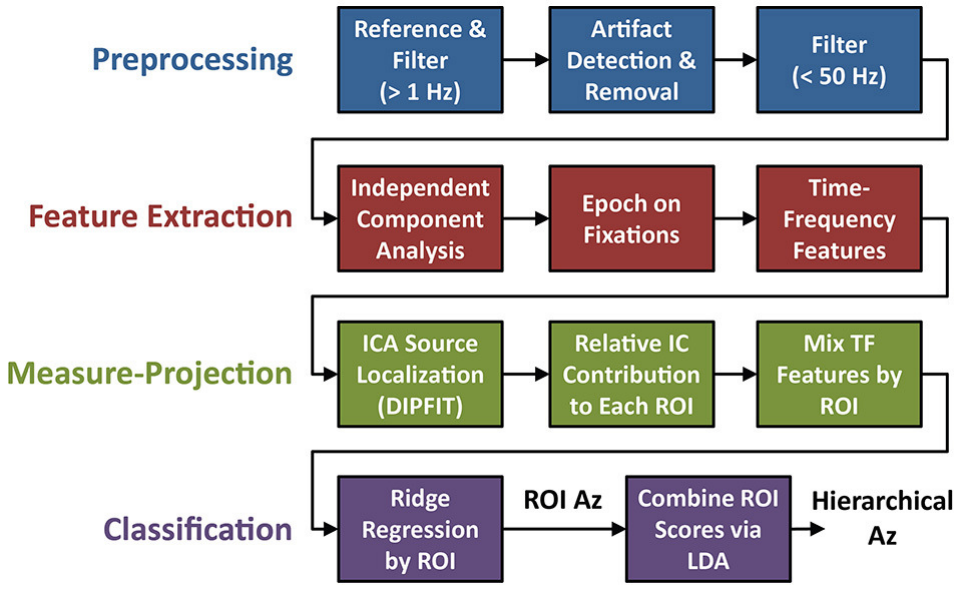
Flowchart of EEG preprocessing and model fitting. The four steps of the analysis include: preprocessing (blue), feature extraction (red), ROI-based measure-projection (green), and hierarchical classification (purple).

Electrophysiological signal acquisition and analysis steps are outlined in Figure 3. EEG recordings were digitally sampled at 1,024 Hz from 64 scalp electrodes over the entire scalp using a BioSemi Active Two system (Amsterdam, Netherlands). External leads were placed on the outer canthi, and above and below the orbital fossa of the right eye to record electrooculography (EOG). EEG was referenced offline to the average mastoids, down-sampled to 256 Hz (*f*_*s*_), and digitally high-pass filtered above 1 Hz using the EEGLAB toolbox (Delorme and Makeig, 2004). Large artifacts were detected using a previous described technique (Touryan et al., 2016). Briefly, EEG sessions were segmented into high-resolution 100 ms epochs, with a 10 ms step size. Epochs were marked as high noise if the average power between 90 and 120 Hz was greater than three standard deviations above the mean for all epochs. These epochs were then removed and the remaining EEG record was lowpass filtered below 50 Hz.

Each “clean” EEG session was decomposed into independent components using the Extended Infomax ICA algorithm implemented in EEGLAB (Delorme and Makeig, 2004). The equivalent dipole locations of these independent sources were then estimated using the EEGLAB implementation of DIPFIT (Scherg, 1990; Pascual-Marqui et al., 1994). IC activation epochs were extracted around each valid fixation using a temporal window spanning 300 ms before and 1,000 ms after fixation onset. Time-frequency features were also calculated for each epoch using a wavelet transform (Torrence and Compo, 1998). Specifically, we used the Morlet wavelet function:

(1) $$\psi_{0}(t)=c\pi^{-1/4}e^{i\omega_{0}t}e^{-t^{2}/2}$$

where ω_0_ is the central frequency and *c* the normalization constant. This function was used to create a basis set of 30 wavelets covering the available frequency range with minimum scale of 2/f_s_ and a discrete step size of 0.25 (wavelet transform software available at http://paos.colorado.edu/research/wavelets/). After the wavelet transform, the spectral power of each epoch was computed via multiplication with the complex conjugate of the corresponding epoch. While this time-frequency decomposition included frequencies from 1 to 128 Hz, only frequencies below 32 Hz were included in subsequent analyses.

To isolate activity in brain regions of interest (ROIs), the above IC activation epochs were linearly mixed based on equivalent dipole location using the initial steps of measure-projection analysis (Bigdely-Shamlo et al., 2013). ICs with equivalent dipoles outside of the MNI model brain volume were identified and excluded from analysis (see Supplementary Section 3). These ICs often corresponded to corneo-retinal potentials (i.e., EOG) or muscle artifacts (i.e., EMG). The remaining *k* IC processes were preserved and their corresponding fixation-locked activation epochs used as the “measure” for each dipole location in the mixing process. Specifically, the fixation-locked activations or measures can be indexed as *M*_*i*_, *i* = 1…*k* for each IC, and the equivalent dipole location *x* ∈ *V* ⊂ *R*^*3*^ indexed as *D*(*x*_*i*_), *i* = 1 …*k*. Importantly, there exists uncertainty in dipole localization arising from errors in tissue conductivity parameters, electrode co-registration, noise in the IC estimate process, and between-subject variability in the location of equivalent functional cortical areas. To capture this uncertainty in the mixing process we can instead model each equivalent dipole as a spherical (3-D) Gaussian with uniform covariance σ^2^, centered at the estimated dipole location *x*_*i*_. The spherical Gaussian is truncated at t*σ to minimize the erroneous influence of distant dipoles in sparsely populated regions. Thus, the probability of dipole *D*(*x*_*j*_) being located at position *y* ∈ *V* now becomes $P_{j}\left(y\right)=TN\left(y;x_{j},\sigma^{2},t\right)$, where *TN* is a truncated normal distribution centered at *x*_*j*_. Then for an arbitrary location *y* ∈ *V*, the expected value of the measure becomes:

(2) $$E\left\{M\left(y\right)\right\}=\langle M\left(y\right)\rangle =\frac{\sum_{i=1}^{k}P_{i}\left(y\right)M_{i}}{\sum_{i=1}^{k}P_{i}\left(y\right)}$$

**Figure 4:**
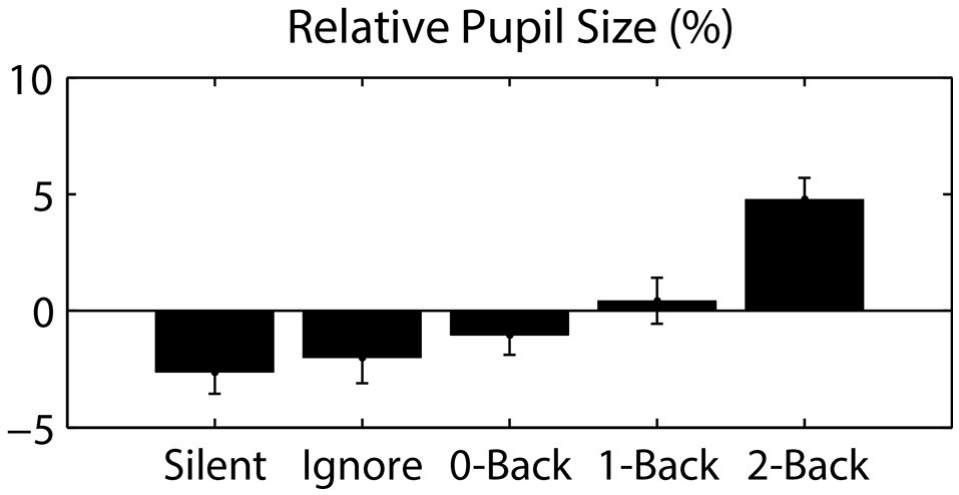
Pupil size, relative to the session average, as a function of auditory task condition. Error bars represent standard error of the mean (SEM).

Where *M(y)* is the combined fixation-locked activity at location *y* from all proximal ICs. We used this approach to calculate the aggregate measure 〈*M*(*y*)〉, either fixation-related potential (FRP) or time-frequency spectrum, for specified regions of the brain volume. For this study, six *a priori* ROIs (Figure 4) were defined using the Measure Projection Toolbox (http://sccn.ucsd.edu/wiki/MPT). Each ROI consisted of all regions of LONI LPBA40 atlas (Shattuck et al., 2008) that included the corresponding anatomical label (e.g., “occipital”). The only additional parameter σ (standard deviation of the Gaussian distribution) in this calculation was set to 12 mm. This value produced smooth spatial distributions in each ROI given the relatively small number of participants (*N* = 14). The six ROIs included: *occipital, fusiform, temporal, parietal, cingulate*, and *frontal* cortices.

### Hierarchical classification

For the classification step, we used a two-stage hierarchical approach to dissociate target and non-target fixation epochs. In the first stage, ridge regression (MATLAB® *ridge* function) was applied separately to the time-frequency epochs from each ROI. Specifically, we applied regularized regression to the entire temporal epoch and frequencies up to 32 Hz. The regularization parameter was determined via calculating the effective degrees of freedom as a function of lambda (λ):

(3) $$df\left(\lambda \right)=tr\left(X{\left(X^{T}X+\lambda I\right)}^{-1}X^{T}\right)=\sum_{j=1}^{m}\frac{d_{j}^{2}}{d_{j}^{2}+\lambda }$$

where *d*_*j*_ are the singular values of the *n x m* data matrix *X*. As these functions were roughly similar across ROIs (Supplementary Figure 3), we selected a hyperparameter value (Lemm et al., 2011) such there were approximately 1 target and 10 non-target observations per degree of freedom (see Model Considerations for a discussion of the validity of this approach). However, the exact value had minimal effect on the results (see Supplementary Section 2). The second stage utilized the regression output, or latent variable estimate, from the six ROIs to provide a single classification score and label for each fixation epoch. In this second stage, we employed linear discriminant analysis (LDA; MATLAB® *fitcdiscr* function) and coefficients for both stages were fit within a single 5-fold cross-validation scheme. Area under the ROC curve (Az) was calculated for each ROI, as well as for the second-stage LDA classifier. Finally, for direct inference into the discriminant neural activity we calculated the forward model for each ROI (Haufe et al., 2014). Specifically, the regression weights (*W*) were used to estimate the forward model (*A*), such that $A=\Sigma_{X}W\Sigma_{\hat{s}}$, where Σ_*X*_ and $\Sigma_{\hat{s}}$ are the empirical data and score covariance respectively.

To facilitate comparison with other approaches, we included two techniques commonly used for single-trial classification of EEG. Both methods were applied directly to the filtered EEG data (64 channels) using the same fixation epochs described above. First, Hierarchical Discriminant Components Analysis (HDCA) was applied with each epoch divided into 8 equal-sized temporal windows (Gerson et al., 2006). Second, we used the xDAWN algorithm (Rivet et al., 2009) to identify the 8 most discriminant spatial filters followed by a Bayesian linear discriminant analysis, collectively referred to as XD+BLDA (Cecotti et al., 2011). Area under the ROC curve was calculated for both of these classifiers on all participants.

## Results

### Behavioral and ocular measures

Detailed behavioral analysis of this study has been previously reported (Ries et al., 2016), however the relevant statistics are summarized below for comparison with the classification results. Reaction time and accuracy were analyzed separately for the visual and auditory tasks using a one-way repeated measures ANOVA (Greenhouse-Geisser correction reported where appropriate). The primary factor was auditory task condition, which had five levels in the visual task (Silent, Ignore, 0-Back, 1-Back, 2-Back), and three levels in the auditory task (0-Back, 1-Back, 2-Back). There was a trend for decreased accuracy in the visual task as a function of cognitive load (i.e., auditory N-back level); however this was not statistically significant (Table 1). We did observe a highly significant effect of cognitive load on reaction time (RT) in the visual task [*F*_(2.73, 35.44)_ = 29.24, *p* < 0.001, η^2^ = 0.69] showing that visual target RT increased as a function of cognitive load. Likewise, analysis of the auditory task showed both a significant decrease in accuracy [*F*_(1.61, 20.96)_ = 6.74, *p* < 0.01, η^2^ = 0.34] and increase in RT [*F*_(1.60, 20.79)_ = 17.64, *p* < 0.001, η^2^ = 0.58] with increasing auditory task demands. While the behavioral results showed that auditory working memory load had a significant negative impact on visual task performance, exhibited through increased RT, the near-ceiling accuracy likely mitigated any decline of this corresponding metric. Together, the behavioral results suggest that participants were not exclusively favoring one modality as performance declined in both the visual and auditory tasks with increased cognitive load.

**Table 1:** ANOVA statistics for accuracy and RT in visual and auditory tasks.

**Factor**	***df***	***F***	***p***	***η^2^***
VISUAL TARGET (BY CONDITION^*^)				
Accuracy	2.67,34.77	2.43	0.088	0.16
RT	2.73,35.44	29.24	<0.001	0.69
AUDITORY TARGET (BY CONDITION^*^)				
Accuracy	1.61,20.96	6.74	0.008	0.34
RT	1.60,20.79	17.64	<0.001	0.58

* *Auditory N-back level: Silent, Ignore, 0-Back, 1-Back, 2-Back*.

Since eye movements were constrained by the nature of the visual task (guided target detection), the majority of ocular metrics did not significantly differ across blocks or conditions. As expected, we found no significant difference in fixation duration (0.962 ± 0.053 s; mean ± STD) or saccade distance (13.316 ± 1.072 degrees) across auditory task conditions. However, we did observe a large change in pupil dilation as a function of cognitive load (Figure 4). Specifically, we calculated the average pupil size in each condition relative to the average size across each participant's entire session. This relative pupil-size metric was significantly modulated by auditory task condition [*F*_(3.04, 39.57)_ = 7.98, *p* < 0.001, η^2^ = 0.38], exhibiting an increase in size with working memory load. Due to static luminance and counterbalanced condition order (see Materials and Methods), this modulation was unlikely to be a consequence of either changes in luminance or time-on-task (Beatty, 1982). Thus, our results indicate that the task-induced cognitive load increased the arousal level of participants, as has been shown in similar paradigms (Kahneman and Beatty, 1966).

### Fixation-related potentials (FRPs) by ROI

**Figure 5:**
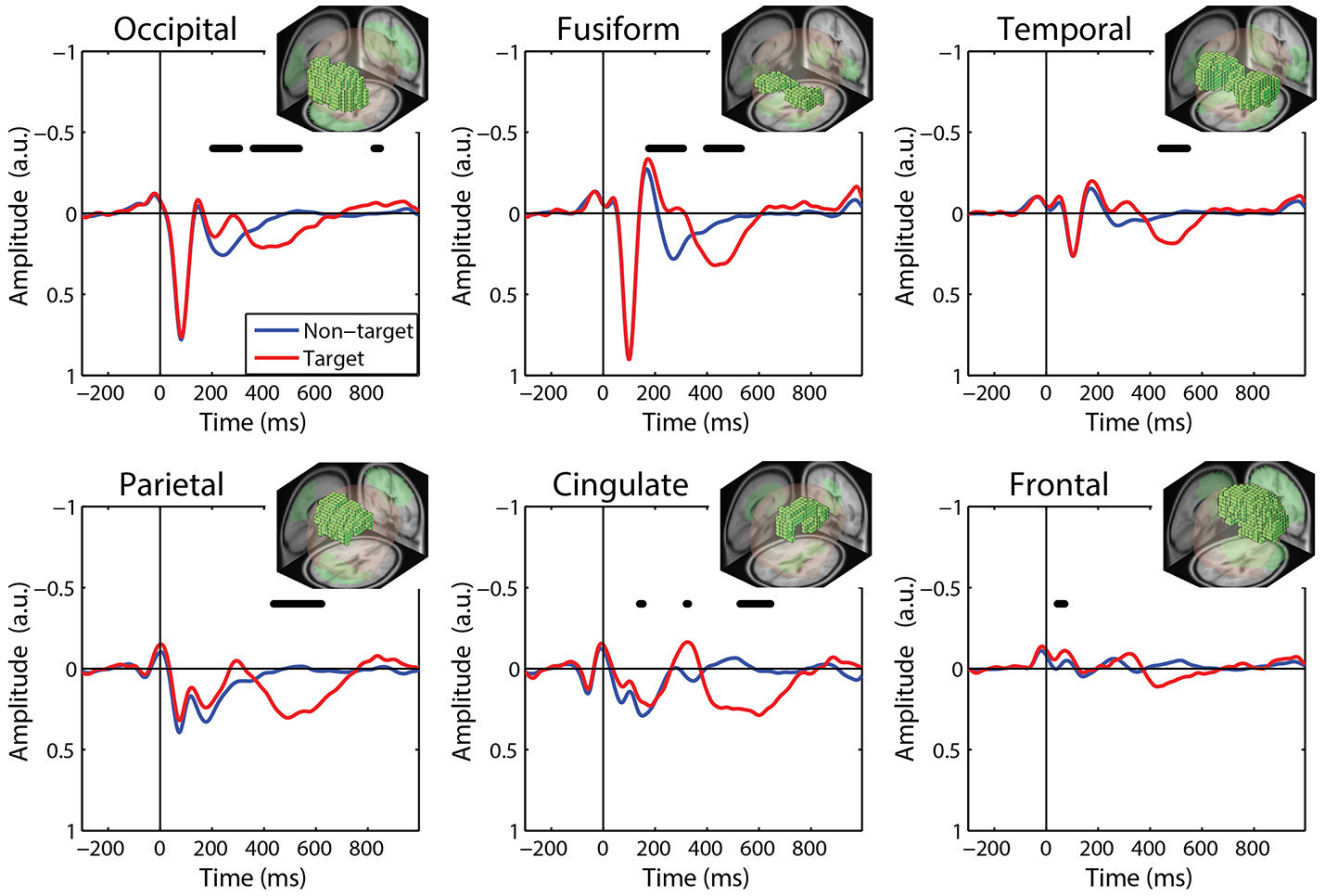
Grand average FRP by ROI. Target and non-target waveforms from each ROI (black line indicates a significant difference, *p* < 0.01). All voxels included in the ROI are shown as the inset. FRP response consists of a linear sum of IC activations weighted by their contribution to the corresponding ROI and are shown in arbitrary units. Note: ICs with equivalent dipoles located outside of the brain volume, such as those produced by EOG, are not aggregated in the ROI FRPs.

We calculated FRPs for each brain region by combining independent components activations within fixation epochs, using a ROI-based measure-projection approach (ROI-MPA). An IC's contribution to a given ROI was determined by the overlap between the anatomically defined region and the equivalent dipole Gaussian density function (see Materials and Methods). Importantly, by excluding equivalent dipoles located outside of this brain volume this approach attempts to minimize the influence of non-brain signals, such as those generated by eye movements, from the ROIs (see Supplementary Section 3). Figure 5 shows the grand average FRPs from each ROI: *occipital, fusiform, temporal, parietal, cingulate*, and *frontal* cortices. To account for the differing number of included ICs, FRPs from each participant were uniformly scaled by total variance and are shown in arbitrary units. All Included epochs were from valid fixations (within 3 degrees of the current stimulus) and free of large artifacts. The average number of target and non-target epochs, by condition, are shown in Table 2.

**Table 2:** Number of epochs included in each condition.

**Condition**	**Target epochs**	**Non-target epochs**
Silent	21 (5)	209 (49)
Ignore	21 (7)	209 (66)
0-Back	23 (5)	212 (40)
1-Back	21 (5)	218 (48)
2-Back	23 (7)	203 (63)
Total	108 (20)	1,051 (211)

*Values represent the mean, standard deviation in parentheses*.

The ROI FRP waveforms shown in Figure 5 exhibit a clear distinction across brain regions. Both target and non-target FRPs show a temporal progression through the visual cortices (occipital, fusiform, temporal) and reflect known electrophysiological signatures, such as the P1 or lambda component. Importantly, the distinction between target and non-target FRPs is evident in most ROIs. To identify periods of significant difference in the FRP waveforms we used a paired *t*-test at causal time points in each ROIs (255 time points × 6 ROIs). A single false-discovery-rate correction for multiple comparisons was then applied to all *p*-values (Benjamini and Hochberg, 1995). As expected, visual cortices show this distinction in earlier epochs, consistent with the visual mismatch negativity (vMMN: 150–250 ms), while the parietal and cingulate cortex exhibit a clear late positive deflection, indicative of the P3 component. In contrast, the frontal cortex shows little saccade-related EOG artifact that would be expected to dominate frontal electrodes (e.g., Fz).

**Figure 6:**
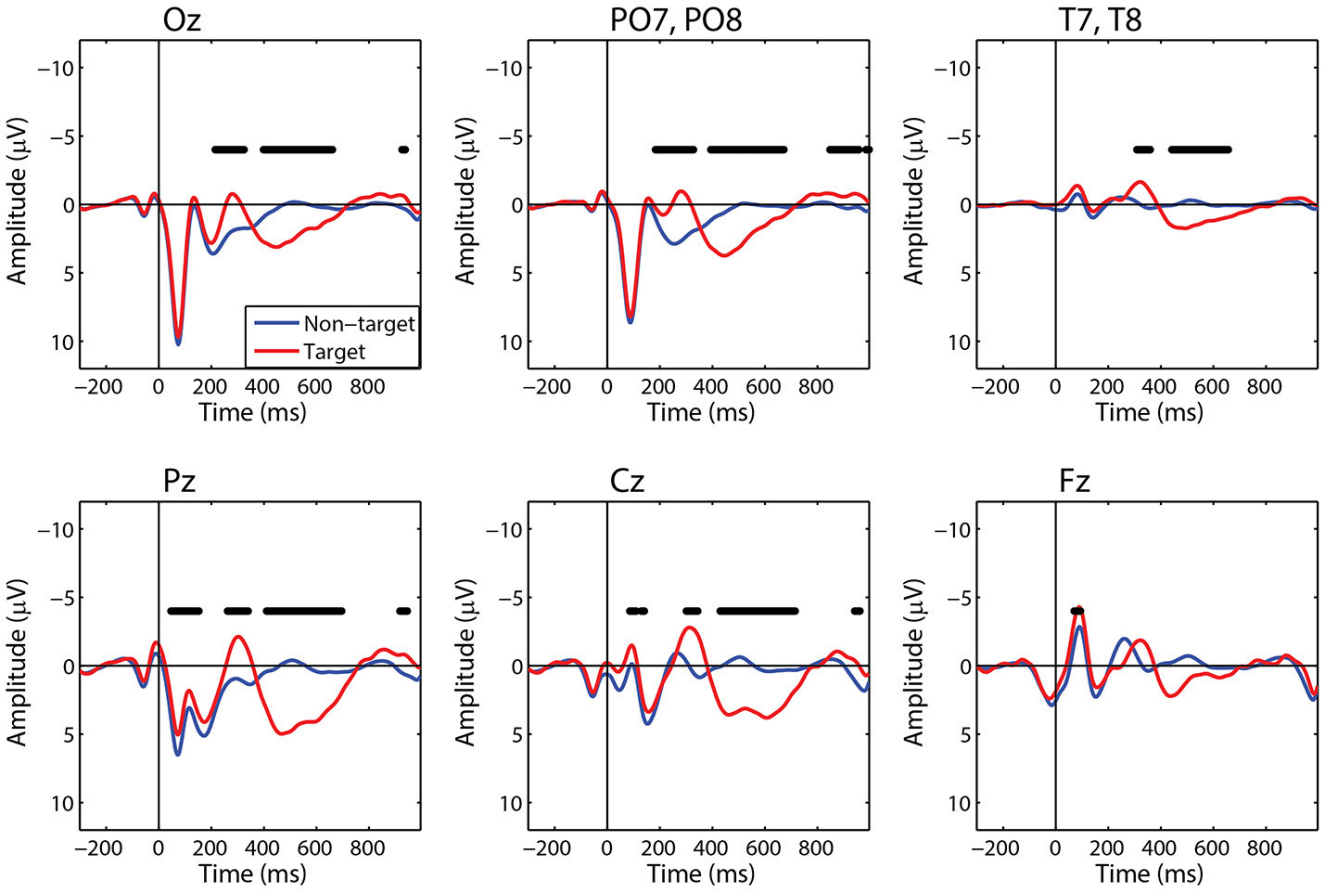
Grand average FRP by electrode. Target and non-target waveforms from electrodes corresponding to each ROI (black line indicates a significant difference, *p* < 0.01).

For comparison to the standard approach, we also calculated target and non-target FRPs for electrodes that most directly correspond to each ROI. Figure 6 shows the grand average FRP from these corresponding electrodes, using the same fixation epochs as above (Table 2). For occipital regions, the electrode and ROI FRPs are quite similar, as these electrodes are least affected by changes in the corneo-retinal potential and other saccade related activity. However, EOG artifact increasingly dominates the anterior regions, especially frontal electrodes (e.g., Fz). This can lead to difficulty in dissociating neural from EOG phenomena in more cognitive processes. For these grand averages, the number of target epochs was an order of magnitude lower than that for non-target epochs. However, a similar result was found for both ROI and electrode FRPs when these numbers were equated by randomly sampling a subset of non-target epochs (Supplementary Figures 1, 2).

Finally, to quantify the effect of auditory task condition (i.e., working memory load) on the ROI FRP we performed the following analysis. We measured the amplitude of the FRP for auditory conditions at either end of the difficulty spectrum: *Ignore* and *2-Back*. These were chosen as representative of low and high cognitive load conditions; although similar results were found when comparing the *Silent* and *2-Back* conditions. To capture the P3 waveform, we calculated the average amplitude within a 300–700 ms post-fixation window. We then applied a two-way repeated measures ANOVA, with factors ROI and condition, to quantify the effect of cognitive load on this components (Table 3). As expected, there was a strong effect of auditory task condition [*F*_(1, 65)_ = 22.45, *p* < 0.001, η^2^ = 0.15] with the amplitude of the P3 being significantly smaller during high, relative to low, working memory load.

**Table 3:** ANOVA statistics for P3 amplitude in the visual task.

**Factor**	***df***	***F***	***p***	***η^2^***
**P3 AMPLITUDE (300–700 ms)**				
Condition^*^	1,65	22.45	<0.001	0.15
ROI	2.13,27.70	11.55	<0.001	0.38
Interaction	5,65	1.69	0.150	0.05

* *Auditory N-back level: Ignore, 2-Back*.

### Classification by ROI

**Figure 7:**
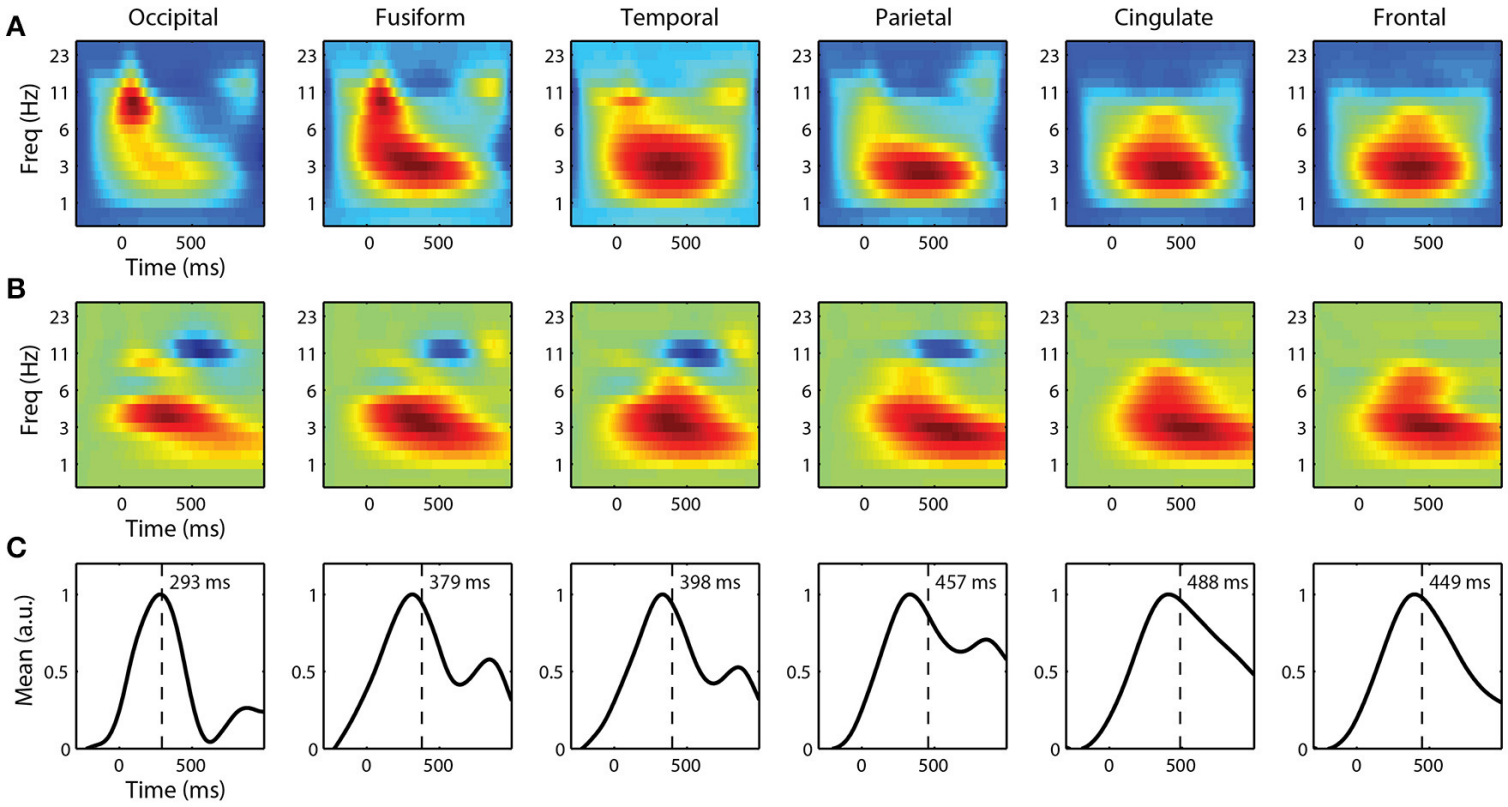
Grand average fixation-related time-frequency features by ROI. **(A)** Average time-frequency features of target epochs. **(B)** Forward model and **(C)** corresponding marginal activation showing the discriminant time course of each ROI. Vertical line represents center-of-mass.

For single-trial classification, we used fixation-locked time-frequency features from each ROI. Before linearly mixing IC activations, we first applied a Morlet wavelet transform to each epoch. We then calculated the spectral power of the wavelet transform before combining these time-frequency epochs. Figure 7A shows the grand average spectral FRPs for target epochs from each ROI. These average time-frequency responses, analogous to event related spectral perturbations (ERSP), show a similar time course as the FRPs above. Visual cortices have an early, mid-frequency (alpha band) component that is the spectral equivalent of the lambda response. Similarly, the parietal, cingulate, and frontal cortices are dominated by a later lower frequency (delta band) activity, reflecting the P3 component.

To classify target from non-target fixation epochs, we used ridge regression on these high-dimensional time-frequency features. We constructed separate classifiers for each ROI that utilized spectral information, below 32 Hz, from the entire fixation epoch. The forward models for each ROI are shown in Figure 7B. Again, the time course is similar to the grand average FRPs, where visual cortices have discriminant activity with smaller latencies and higher-frequency components. The marginal activations (Figure 7C) provide a more direct view of the temporal profile of the discriminant activity.

**Figure 8:**
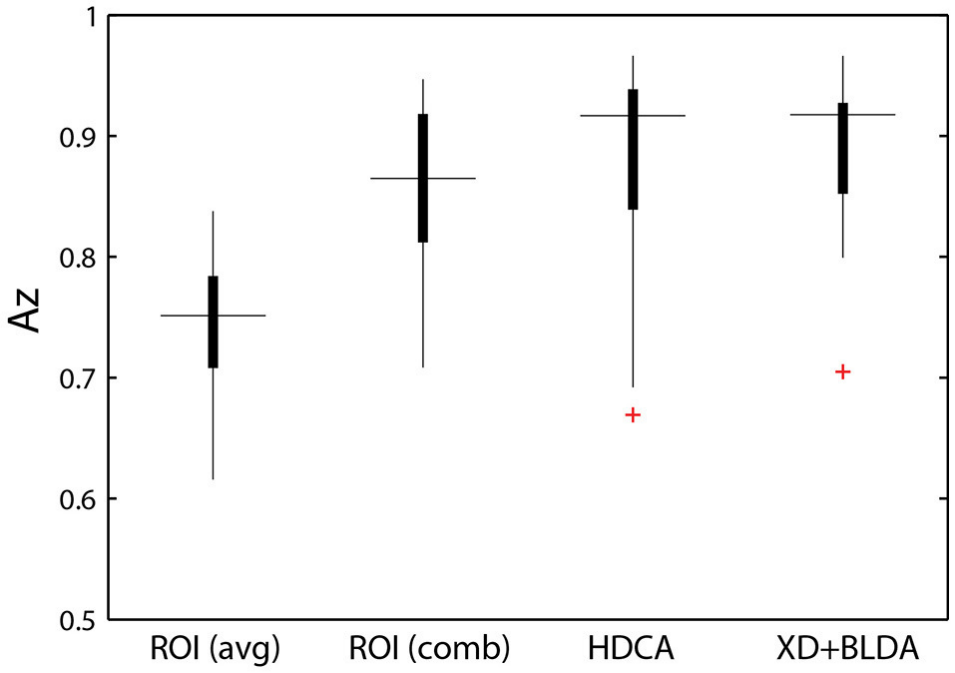
Classifier performance. ROI classifier performance (averaged) compared with the hierarchical approach (combined). Also shown are channel-based classification techniques HDCA and XD+BLDA. Horizontal lines indicate median values and box area covers the 25th–75th percentiles.

The relative discriminant power of each ROI was quantified by using classifier performance in a two-way repeated measures ANOVA, with factors ROI and auditory task condition (Table 4). We found a significant modulation of the area under the ROC curve (Az) by region [*F*_(2.41, 156.73)_ = 12.84, *p* < 0.001, η^2^ = 0.15]. The average performance across all ROIs and participants was 0.741 ± 0.068 (Figure 8), substantially below behavioral performance in the visual detection task (average accuracy = 0.974 ± 0.030).

**Table 4:** ANOVA statistics for classifier performance in the visual task.

**Factor**	***df***	***F***	***p***	***η^2^***
**ROI CLASSIFIER PERFORMANCE (Az)**				
Condition^*^	2.41,156.73	12.84	<0.001	0.15
ROI	2.40,124.59	1.89	0.147	0.03
Interaction	8.94,116.23	1.07	0.388	0.06
**HIERARCHICAL CLASSIFIER PERFORMANCE (Az)**				
Condition^*^	2.04,26.55	1.16	0.329	0.08
**HIERARCHICAL CLASSIFIER SCORE (TARGET)**				
Condition^*^	2.66,34.60	3.63	0.026	0.22

* *Auditory N-back level: Silent, Ignore, 0-Back, 1-Back, 2-Back*.

Integration across regions required a second-stage classifier applied to the output of the ROI regression step. For each epoch, the output from the ROI classifiers (i.e., vector of six classification scores) were combined using a linear discriminant function. Not surprisingly, this hierarchical approach resulted in significantly better performance (Az: 0.851 ± 0.096) than the individual ROI classifiers (*p* < 0.001; Wilcoxon signed rank test). Interestingly, there was a wide range in classifier performance across participants with Az values ranging from 0.708 to 0.947, indicating that for some individuals our approach was able to identify visual targets at an accuracy similar to behavioral performance. This was despite ongoing neural activity related to the concurrent auditory task as well as the planning and execution of eye movements.

This hierarchical approach compared favorably to other common classification techniques (Figure 8B). Specifically, we applied Hierarchical Discriminant Components Analysis (Gerson et al., 2006) to the EEG channel data using the same epochs as above. We also applied the xDAWN filtering algorithm (Rivet et al., 2009) followed by Bayesian linear discriminant analysis, or XD+BLDA (Cecotti et al., 2011). HDCA and XD+BLDA classification accuracies were similar to our hierarchical approach with HDCA having slightly higher overall performance (*p* = 0.013).

**Figure 9:**
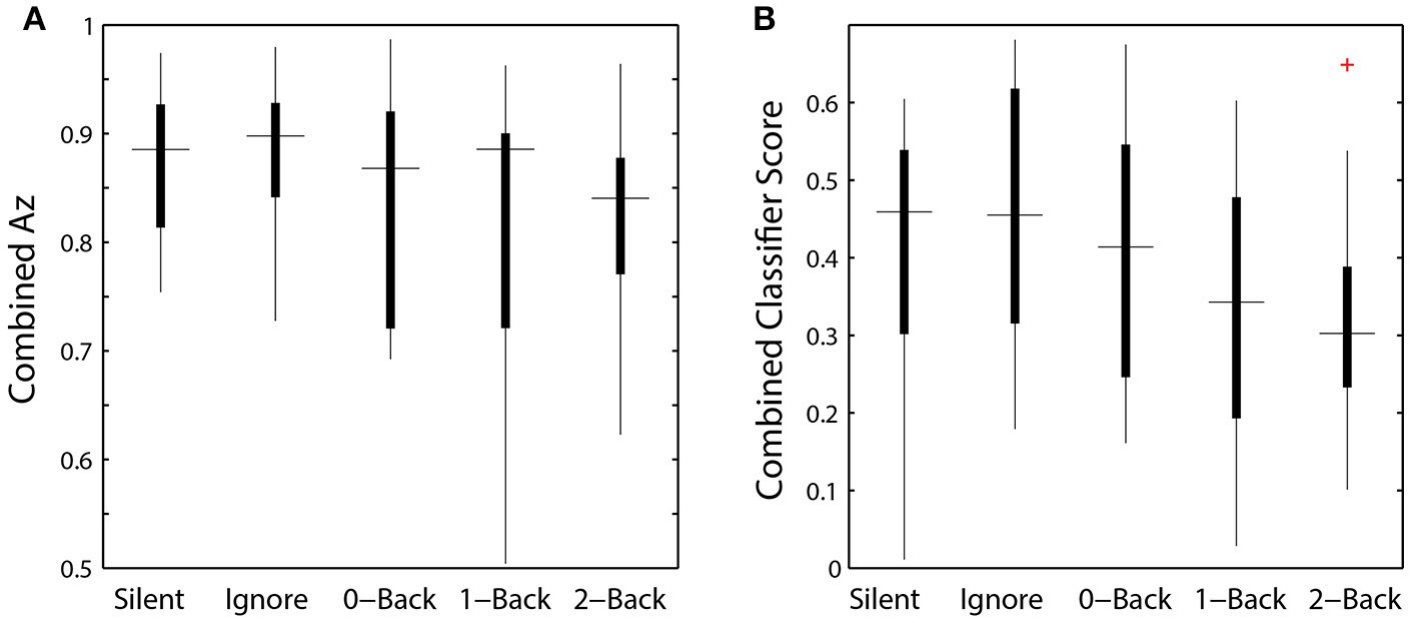
Hierarchical classifier performance and task difficulty. **(A)** Classifier performance as a function of auditory task condition. **(B)** Classifier target scores as a function of auditory task condition. Horizontal lines indicate median values and box area covers the 25th–75th percentiles.

While we were able to classify visual target from non-target stimuli during a concurrent auditory task, there was a significant modulation of ROI classification performance as a function of cognitive load (Table 4). Here, this modulation was the inverse of that observed in the relative pupil size. Classification performance decreased with increasing auditory task demands; except in the silent and ignore condition in which performance was similar. At the hierarchical stage, while the target scores were significantly modulated by condition (Figure 9B), the classification performance was not (Table 4, Figure 9A). Much like behavioral accuracy in the visual task, the hierarchical classifier performance remained relatively constant across auditory task conditions. However, increasing working memory load clearly affected both the neural activity and pupil diameter in a manner consistent with increased arousal (Murphy et al., 2011).

## Discussion

In this study, we used a novel approach to determine if the neural response associated with visual target detection could be separated into meaningful components in the presence of eye movements and concurrent task demands. Here, we employed a single framework to isolate neural from non-neural activity and to separate the FRP into cortical regions. Using common statistical techniques we were then able to classify target from non-target FRPs across ROIs on a single-trial basis at a level similar to state-of-the-art machine learning algorithms. By doing so, we were able to show a clear time-course of discriminant activity associated with target detection as well as the modulating effect of cognitive load. While our task design mitigated the overlapping response from previous or subsequent fixations, the results demonstrate the potential for separating task-relevant neural activity in more complex contexts that include eye movements and concurrent tasks.

The EEG analysis framework described here is both specific enough to separate activity by ROI and sensitive enough to evaluate the effects of cognitive load. While more traditional channel-based approaches of FRP analysis may be able to separate these effects by scalp location (e.g., Oz vs. Pz), the inference into the constituent neural sources remains more difficult. Likewise, channel-based approaches require an explicit EOG mitigation or removal process, using ICA (Plöchl et al., 2012) or other techniques (Parra et al., 2005). For example, a number of recent studies have utilized ICA for the identification and removal of EOG components (Nikolaev et al., 2011, 2013; Devillez et al., 2015). However, these studies typically included a manual or semi-manual step for the identification of ICs related to corneo-retinal potentials and eyelid artifacts (although see Mognon et al., 2011; Plöchl et al., 2012). In contrast, our approach uses equivalent dipole locations to include or exclude particular ICs. While there is ongoing debate as to the accuracy of source localization techniques such as LORETA, there is growing evidence that suggests independent sources are indeed dipolar (Delorme et al., 2012). Fortunately, eye movement related ICs typically explained large fractions of the total signal variance and resolve to equivalent dipoles outside the brain volume with relatively little residual error.

In comparison, IC clustering results (Makeig et al., 2002) are highly dependent on the choice of the clustering parameters (in many cases up to 12 tunable parameters without a clear physiological interpretation for each parameter) and provide no guarantees in terms of producing clusters at particular ROIs. However, the ROI-based measure projection approach (ROI-MPA) is able to focus the analysis on selected ROIs while utilizing only a single parameter with a physiological interpretation (i.e., the expected spatial uncertainty in IC dipole localization). Likewise, IC clusters are not well-suited for single-trial analysis. Simply averaging the single-trial activity of the ICs contained in each cluster would not properly account the spatial distribution of dipole locations. For example, ICs adjacent to the cluster boundary would be excluded (weighted zero) while ICs just inside the cluster weighted at unity. ROI-MPA directly incorporates this spatial information and the ROI structure by forming a weighted sum based on IC spatial probability overlap with each ROI.

### Eye tracking, pupillometry, and EEG

A growing number of studies are combining eye tracking with EEG to enable the exploration for neural activity during visual search. While the task employed here was not a visual search paradigm, our results demonstrate the ability to acquire and utilize gaze position to detect saccades and quantify evoked neural activity. Importantly, our experimental configuration employed a head-free tracking system (SMI RED 250) without a requisite chinrest. This configuration facilitates visual search paradigms or related tasks requiring a large field of view that may naturally engender small head movements. To improve the spatial accuracy of such a system, our analysis included a *post-hoc* calibration of gaze position. Specifically, we utilized task information to infer gaze position when adjusting the offline calibration model. While this type of information may not always be available, experimenters can and should use an opportunistic calibration approach during periods where gaze position can reasonably be inferred (e.g., prior to trial initiation or visual target detection).

An additional benefit derived from the inclusion of eye tracking is the coincident measure of pupil size. For example, the change in pupil diameter shown here suggests that increased working memory load resulted in an increase in arousal level, an important modulator of cognitive performance. Since arousal is largely regulated through the norepinephrine system via the locus coeruleus (LC), a nucleus within the dorsal pons, it cannot be measured directly via EEG. However, several studies have shown that pupil dilation can be used as a proxy for LC activity (Aston-Jones and Cohen, 2005; Murphy et al., 2011; Hong et al., 2014). Furthermore, the LC receives input from anterior cingulate and dorsolateral prefrontal cortex and some studies have suggested that the LC system underlies the parietal P3 ERP, specifically the P3b (Nieuwenhuis et al., 2005, 2011).

Overall, our results confirm the localization of the P3 to parietal ROIs and show a significant effect of arousal on both pupil diameter and P3 amplitude (Murphy et al., 2011). In addition, target FRPs in the posterior ROIs show a negative deflection, relative to non-target FRPs, beginning around 200 ms post-fixation. This difference is consistent with the visual mismatch negativity (vMMN); a negative posterior deflection elicited by an infrequent (deviant) visual stimulus presented in a homogenous sequence of frequent (standard) stimuli (Czigler et al., 2002). In particular, this difference is consistent with the later components of the vMMN associated with memory-comparison-based change detection (Kimura et al., 2009). However, since the infrequent stimuli (Ts) are also task-relevant, it is difficult to dissociate this vMMN from the attentional orienting component of the P3 (Polich, 2007).

Interestingly, the frontal ROI and Fz electrode showed a small but significant difference between target and non-target fixations at an early latency (approximately 80 ms). Previous studies have shown activity associated with peripheral detection in frontal-parietal regions early in and even prior to target fixations (Dias et al., 2013; Devillez et al., 2015). In this paradigm, target stimuli were never immediately adjacent to the current grid location (red annulus), making the peripheral detection of an upcoming target unlikely. However, it would be reasonable for participants to anticipate a target fixation after a sequence of non-target stimuli were encountered. This phenomenon illustrates the manifold difficulties in the interpretation of eye movement related activity given the dependencies between eye movement behavior (e.g., saccade size, fixation duration) and the elicited response. Similarly, in free-viewing contexts there remains the additional challenge of separating overlapping responses from adjacent saccades and fixations. Fortunately, several techniques have now been proposed to address this potential confound using regression or GLM-based approaches (Burns et al., 2013; Smith and Kutas, 2015; Kristensen et al., 2017).

### Model considerations

The ICA-based, hierarchical classification algorithm described here is not ideally suited for real-time application or meant as an alternative to other channel-based approaches (e.g., HDCA). Rather, the goal of this study was to identify the discriminant neural response in each ROI and to quantify the effect of cognitive load on that response. As such, we did not take additional steps to separate data in our cross-validation scheme. Due to data limitations, ICA was applied to the entire EEG record for each participant rather than independently to each training set. Since ICA is an unsupervised technique, however, the potential for overfitting is limited. Additionally, we selected the hyperparameter by balancing the effective degrees of freedom with the number of data points (fixation epochs) rather than through a separate cross-validation step. Here, the exact choice of parameter did not substantially influence the results (see Supplementary Section 2). The added separation of these additional cross-validation steps would significantly reduce the amount of data and the quality of the estimated discriminate functions without providing any additional insight into the neural processes.

### Implications for BCI

Importantly, our hierarchical classification scheme was shown to perform at a level similar to other state-of-the-art machine learning algorithms such as HDCA and XD-BLDA. This result suggests that our approach captured the majority of the task-related variance within the EEG record. While the average FRPs are useful and exhibit an effect of cognitive load, single-trial classification techniques can reveal additional discriminative activity (Brouwer et al., 2012). The forward model of each ROI (Figure 7) reveal both the time course and spectral characteristics of the discriminant neural response. Thus, while our approach imposes an additional computational burden, compared with the above methods, it adds insight into the source of task-related neural activity.

Our results can likewise be used to guide BCI development and future applications. P3-based paradigms remain a key component of the BCI application space, such as the P300 Speller (Krusienski et al., 2006). Presently, these reactive BCIs typically classify the neural response to passively viewed stimuli, such as in a rapid serial visual presentation (Gerson et al., 2005; Touryan et al., 2011; Bigdely-Shamlo et al., 2013). In this passive condition, stimuli are presented to the user who detects the desired target (e.g., target object within an image). In contrast, for targets occurring in natural or ordered environments, a more ecologically valid approach for detection would be through goal-directed visual search (Jangraw et al., 2014; Ušćumlić and Blankertz, 2016). In this case, stimulus presentation is controlled through the user's search strategy, with fixation onset serving as a natural time-locking event. Thus, the growing body of work on single-trial classification of FRPs will support the improved performance of future FRP BCI technology.

## Conclusion and future work

In this study, we provide a principled framework for interpreting EEG in the presence of eye movements and concurrent task demands by adapting a recently developed independent source aggregation technique (Bigdely-Shamlo et al., 2013). This approach enabled us to both quantify the discriminant information contained within each cortical region and measure the effect of cognitive load on the evoked response. While these phenomena have been previously observed, our results demonstrate the feasibility and utility of combining synchronous recordings of EEG and eye-tracking to measure both sensory and cognitive processes. Our approach can be extended to tasks that incorporate unconstrained eye movements, however, additional techniques would be needed to account for the overlapping activity from adjacent saccades and fixations.

In this experiment we did not explicitly manipulate top-down (goal-directed) or bottom-up (stimulus driven) components of the visual task beyond increasing the overall working memory load. However, our ROI mapping framework would be well suited for such an assessment. ROI analysis could be applied across conditions to identify what factors bias top-down vs. bottom-up neural activity in a visual search paradigm. Eye movements biased by top-down task influences may have greater pre- or post-saccadic activity in frontal cortices (Nikolaev et al., 2013). Likewise, eye movements driven by bottom-up stimulus influences may have greater post-saccadic activity in occipital cortex. This distinction may become essential for understanding free-viewing search in natural scenes where visual information leading to a detection event can be accumulated across fixations (Jangraw et al., 2014) rather than isolated to a single gaze position.

## Ethics statement

The voluntary, fully informed consent of the persons used in this research was obtained in written form. The document used to obtain informed consent was approved by the U.S. Army Research Laboratory's Institutional Review Board (IRB) in accordance with 32 CFR 219 and AR 70-25, and also in compliance with the Declaration of Helsinki. The study was reviewed and approved (approval# ARL 14-042) by the U.S. Army Research Laboratory's IRB before the study began.

## Author contributions

JT, AR, and PC develop the task and conducted the experiment. VL and NB developed the single-trial ROI method. AR and JT analyzed the data. JT wrote the manuscript.

### Conflict of interest statement

The authors declare that the research was conducted in the absence of any commercial or financial relationships that could be construed as a potential conflict of interest.
