# Supplementary material for: Isolating Discriminant Neural Activity in the Presence of Eye Movements and Concurrent Task Demands
Source: Front Hum Neurosci. 2017 Jul 7;11:357. doi: 10.3389/fnhum.2017.00357 (PMC5501009; doi:10.3389/fnhum.2017.00357)
Supplement: Supplementary file 1 [file DataSheet1.DOCX]

***Supplementary Material***

**Isolating Discriminant Neural Activity in the Presence of Eye Movements and Concurrent Task Demands**

Jon Touryan^[[1]](#footnote-1)^*, Vernon J. Lawhern^1^, Patrick M. Connolly^2^, Nima Bigdely-Shamlo^3^, and Anthony J. Ries^1^

^1^U.S. Army Research Laboratory, Aberdeen Proving Ground, MD 21005, USA

^2^Teledyne Scientific Company, Durham, NC 27703, USA

^3^Qusp Labs, San Diego, CA 92121

*** Correspondence:** Dr. Jon Touryan, U.S. Army Research Laboratory, Aberdeen Proving Ground, MD 21005, USA; e-mail: [jonathan.o.touryan.civ@mail.mil](mailto:jonathan.o.touryan.civ@mail.mil)

1. **Matched Epoch Numbers**


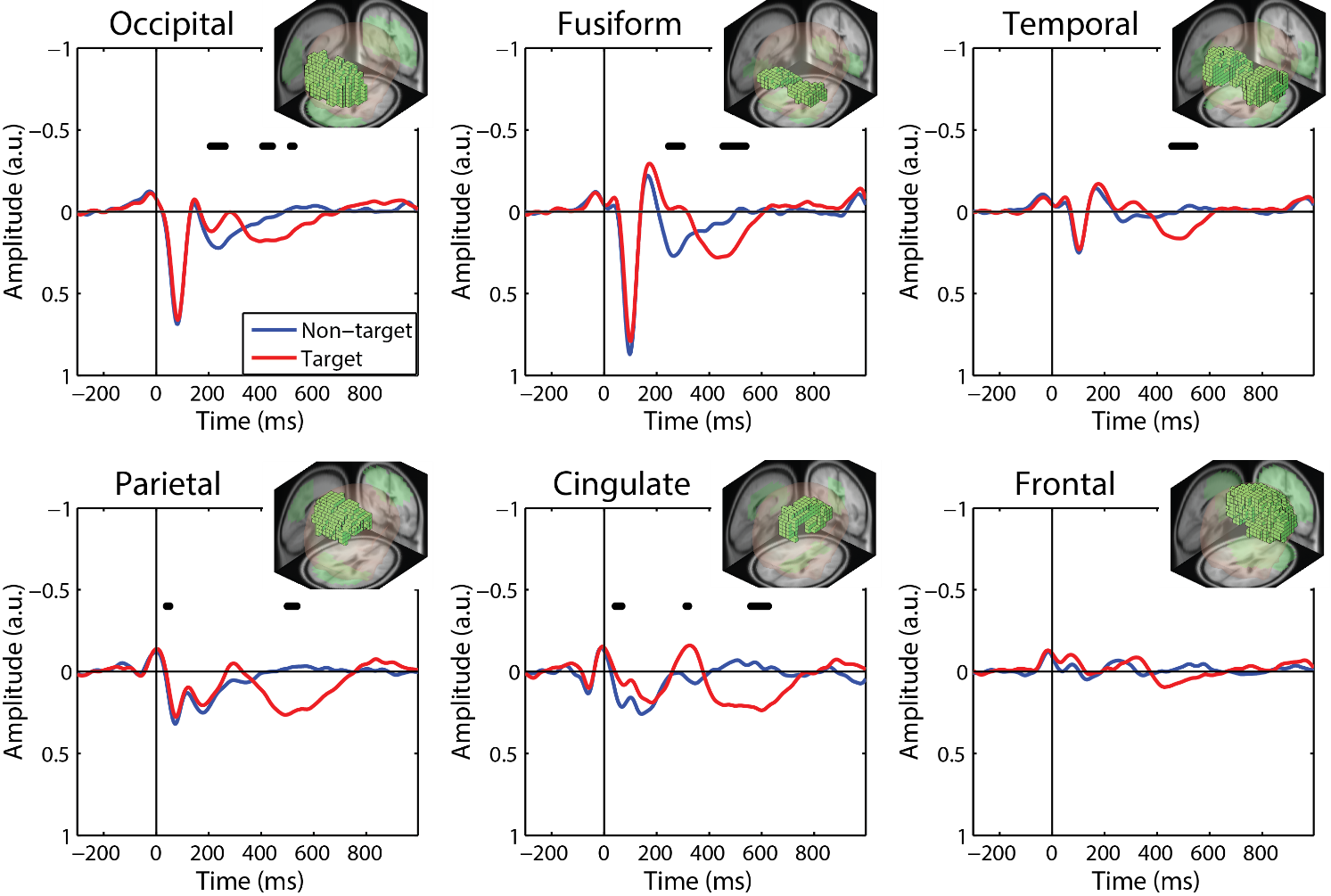


**Supplementary Figure 1:** Grand average FRP by ROI. Target and non-target waveforms from each ROI (black line indicates a significant difference, *p* < 0.01). The number of target and non-target epochs were matched for each subject by randomly selecting a subset of non-target epochs.

**
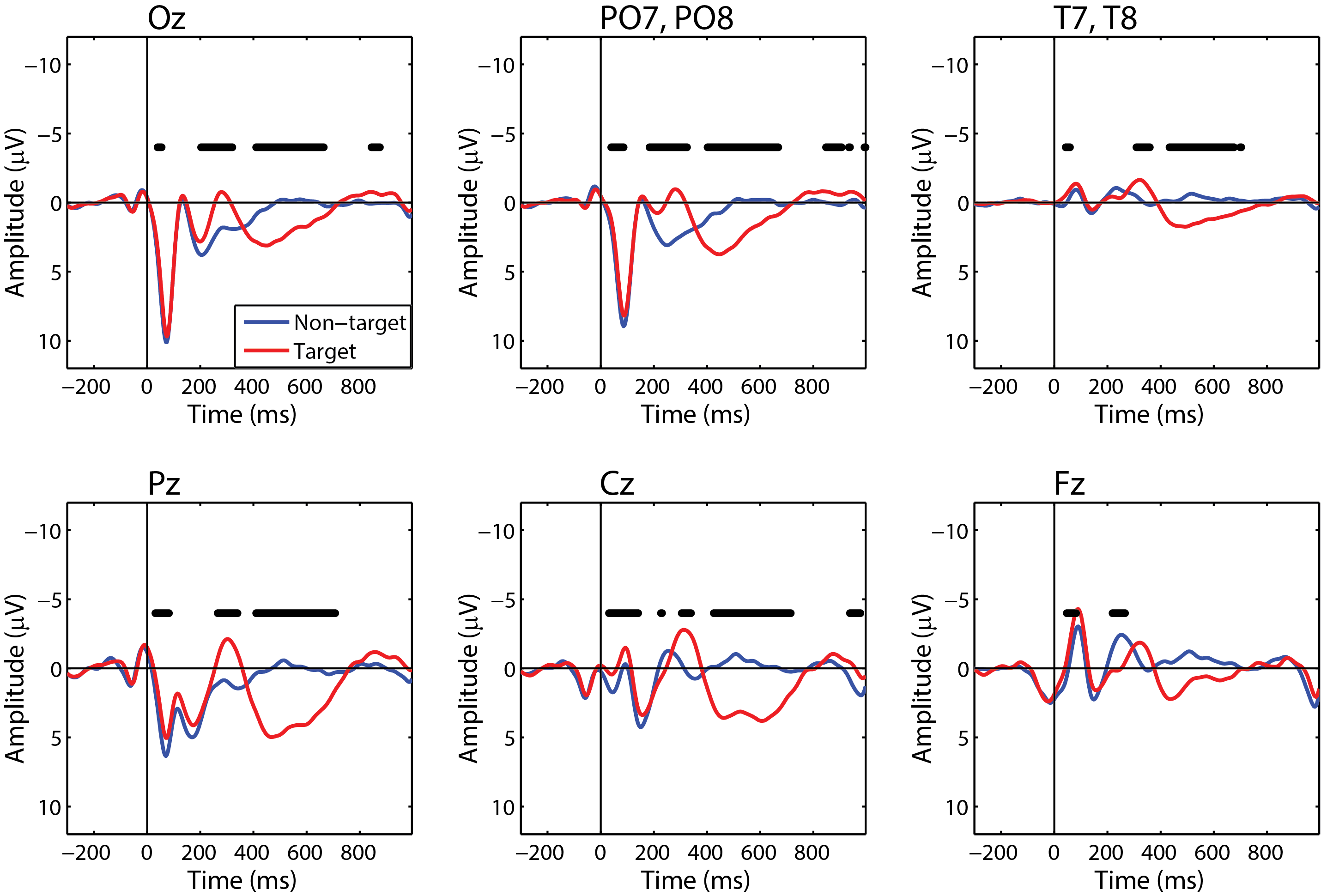
**

**Supplementary Figure 2:** Grand average FRP by electrode. Target and non-target waveforms from electrodes corresponding to each ROI (black line indicates a significant difference, p < 0.01). The number of target and non-target epochs were matched for each subject by randomly selecting a subset of non-target epochs.

1. **Hyperparameter Selection**


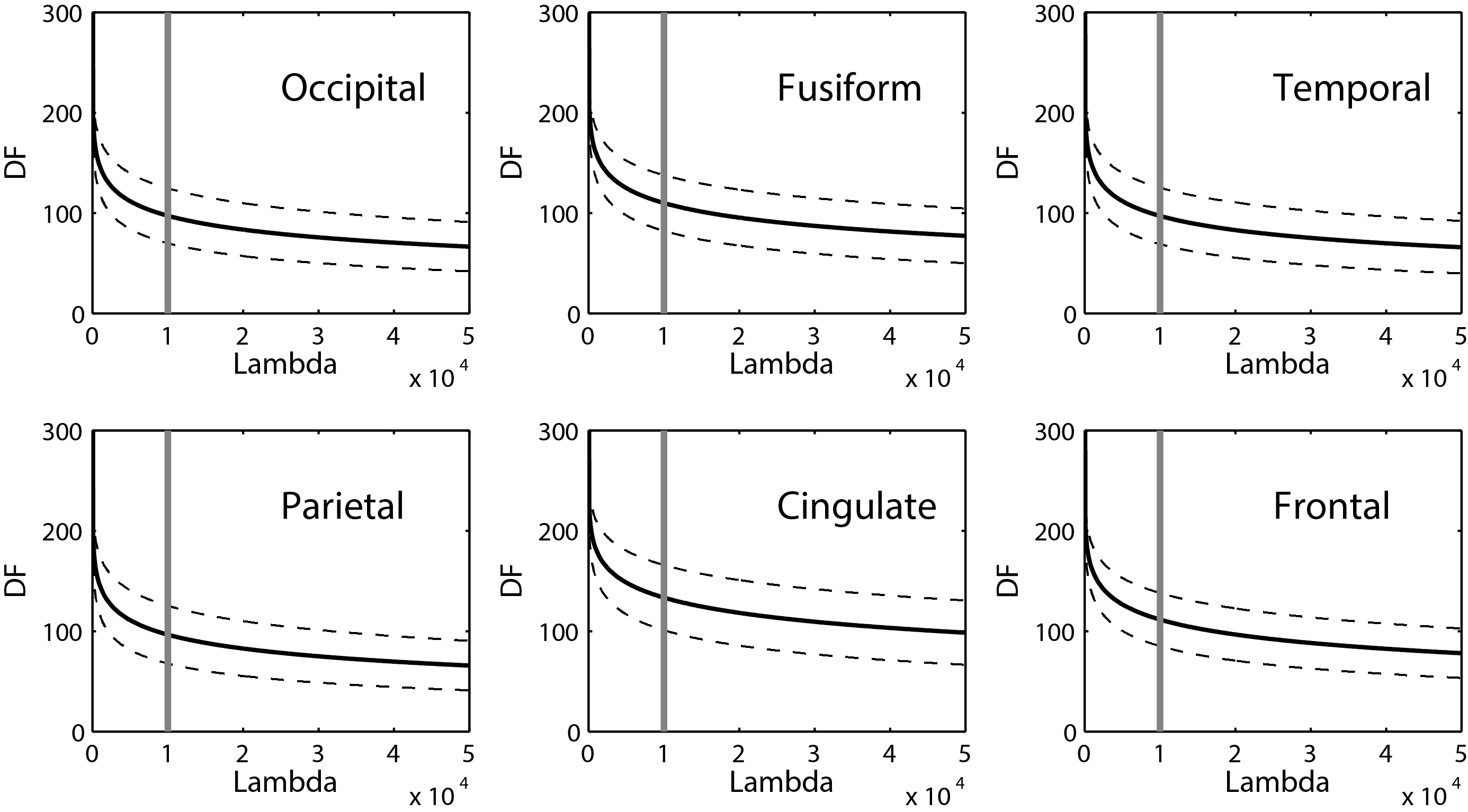


**Supplementary Figure 3:** Estimated degrees of freedom as a function or regularization. Degrees of freedom (DF) decrease with an increase in the regularization parameter (lambda). Horizontal line indicates the selected value lambda resulting in approximately 10 samples per degree of freedom.

The relative discriminant power of each ROI was quantified by using classifier performance in a two-way repeated measures ANOVA, with factors ROI and auditory task condition. Two one-way ANOVA’s were used to quantify the performance of hierarchical classifier and modulation of the corresponding target-epoch score. The results of these ANOVA are shown for lambda values of 1,000 (**Table SI**) and 50,000 (**Table SII**). These results are similar to **Table IV**, showing an effect of task condition on ROI (stage 1) but not hierarchical (stage 2) classifier performance.

**Table SI**

ANOVA statistics for classifier performance in the visual task (lambda = 1,000).

|  | **Factor** | ***df*** | ***F*** | ***p*** | ***η^2^*** |
| --- | --- | --- | --- | --- | --- |
| **ROI Classifier Performance (Az)** | | | | |  |
|  | Condition* | 2.54,165.06 | 9.12 | <0.001 | 0.11 |
|  | ROI | 2.47,128.27 | 1.25 | 0.293 | 0.02 |
|  | Interaction | 8.11,105.46 | 0.87 | 0.544 | 0.05 |
| **Hierarchical Classifier Performance (Az)** | | | | | |
|  | Condition* | 2.17,28.21 | 1.04 | 0.373 | 0.07 |
| **Hierarchical Classifier Score (Target)** | | | | | |
|  | Condition* | 2.68,34.88 | 3.55 | 0.028 | 0.21 |

* Auditory N-back level: Silent, Ignore, 0-Back, 1-Back, 2-Back

**Table SII**

ANOVA statistics for classifier performance in the visual task (lambda = 50,000).

|  | **Factor** | ***Df*** | ***F*** | ***p*** | ***η^2^*** |
| --- | --- | --- | --- | --- | --- |
| **ROI Classifier Performance (Az)** | | | | |  |
|  | Condition* | 2.47,160.36 | 14.61 | <0.001 | 0.16 |
|  | ROI | 2.51,130.68 | 1.94 | 0.136 | 0.03 |
|  | Interaction | 7.56,98.34 | 1.45 | 0.188 | 0.08 |
| **Hierarchical Classifier Performance (Az)** | | | | | |
|  | Condition* | 2.13,27.75 | 1.10 | 0.352 | 0.08 |
| **Hierarchical Classifier Score (Target)** | | | | | |
|  | Condition* | 2.55,33.10 | 3.47 | 0.033 | 0.21 |

* Auditory N-back level: Silent, Ignore, 0-Back, 1-Back, 2-Back

1. **Independent Components Excluded from Analysis**

**
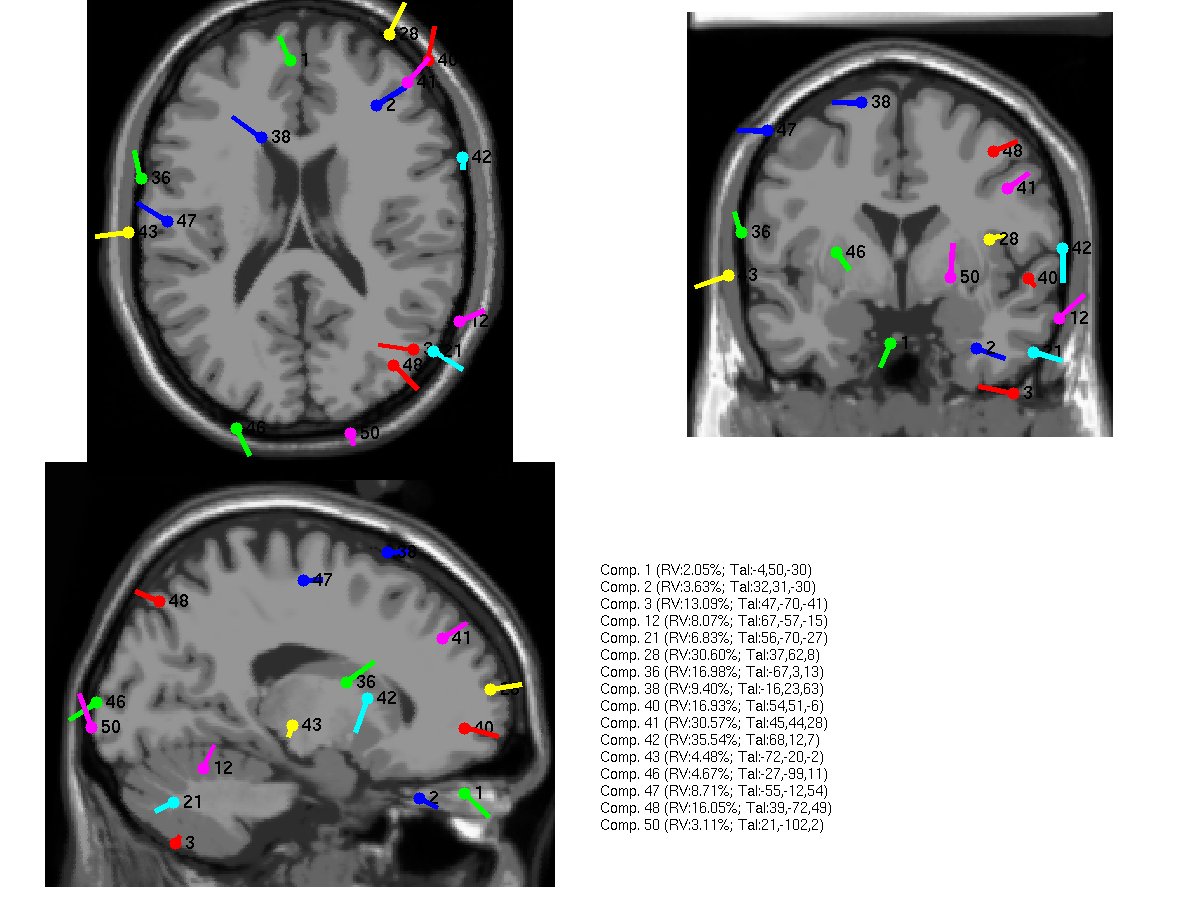

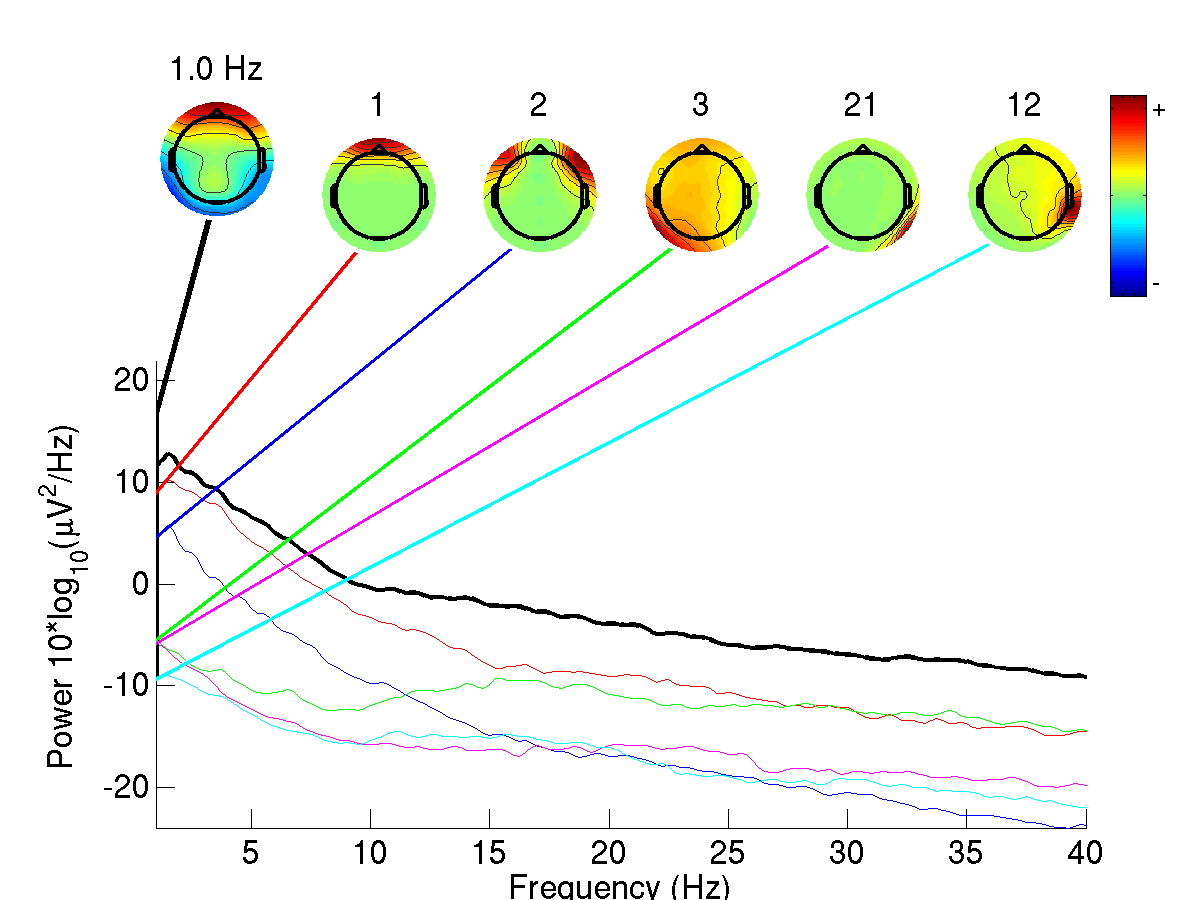
**

**Participant 2:** (left) Equivalent dipole locations of all excluded independent components. (right) Scalp map and power spectra of the first five excluded components.

**
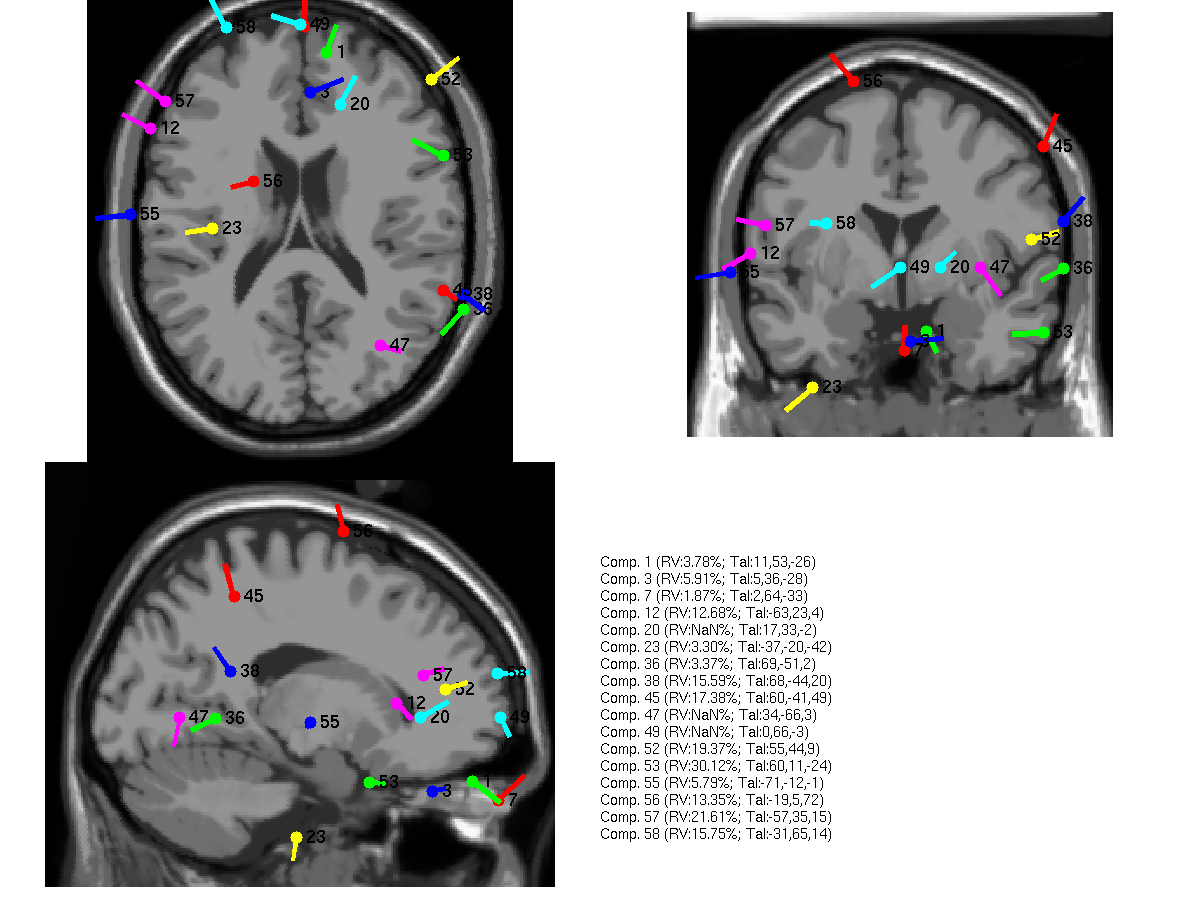
** **
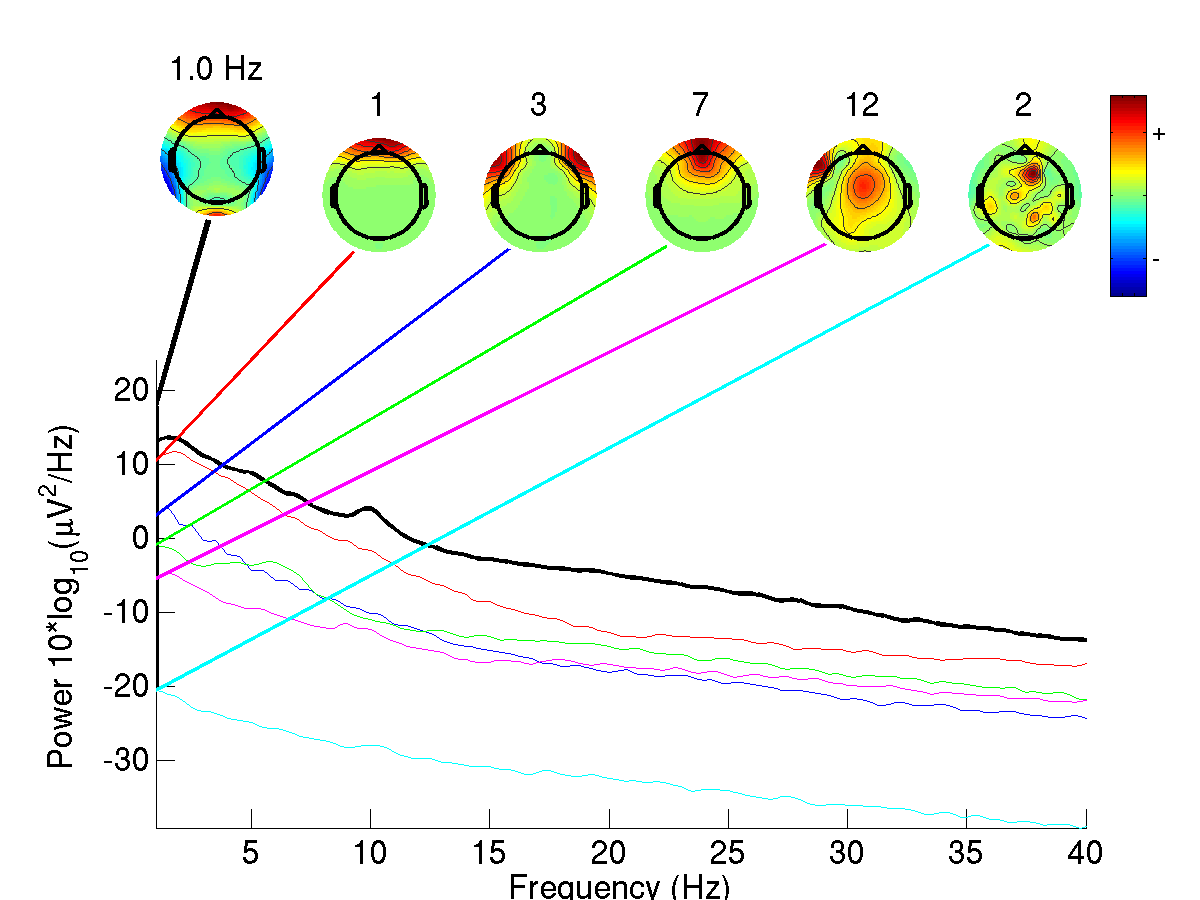
**

**Participant 1:** (left) Equivalent dipole locations of all excluded independent components. (right) Scalp map and power spectra of the first five excluded components.

**
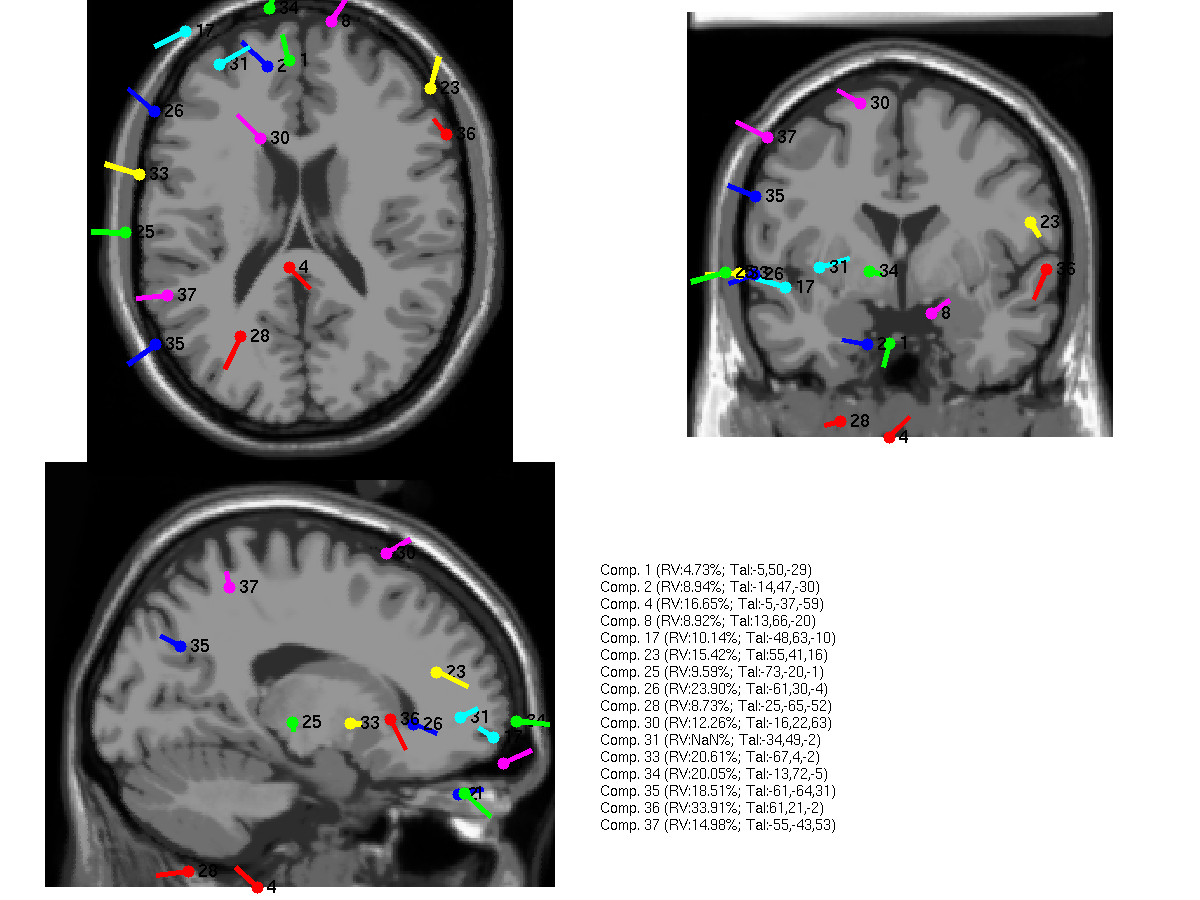

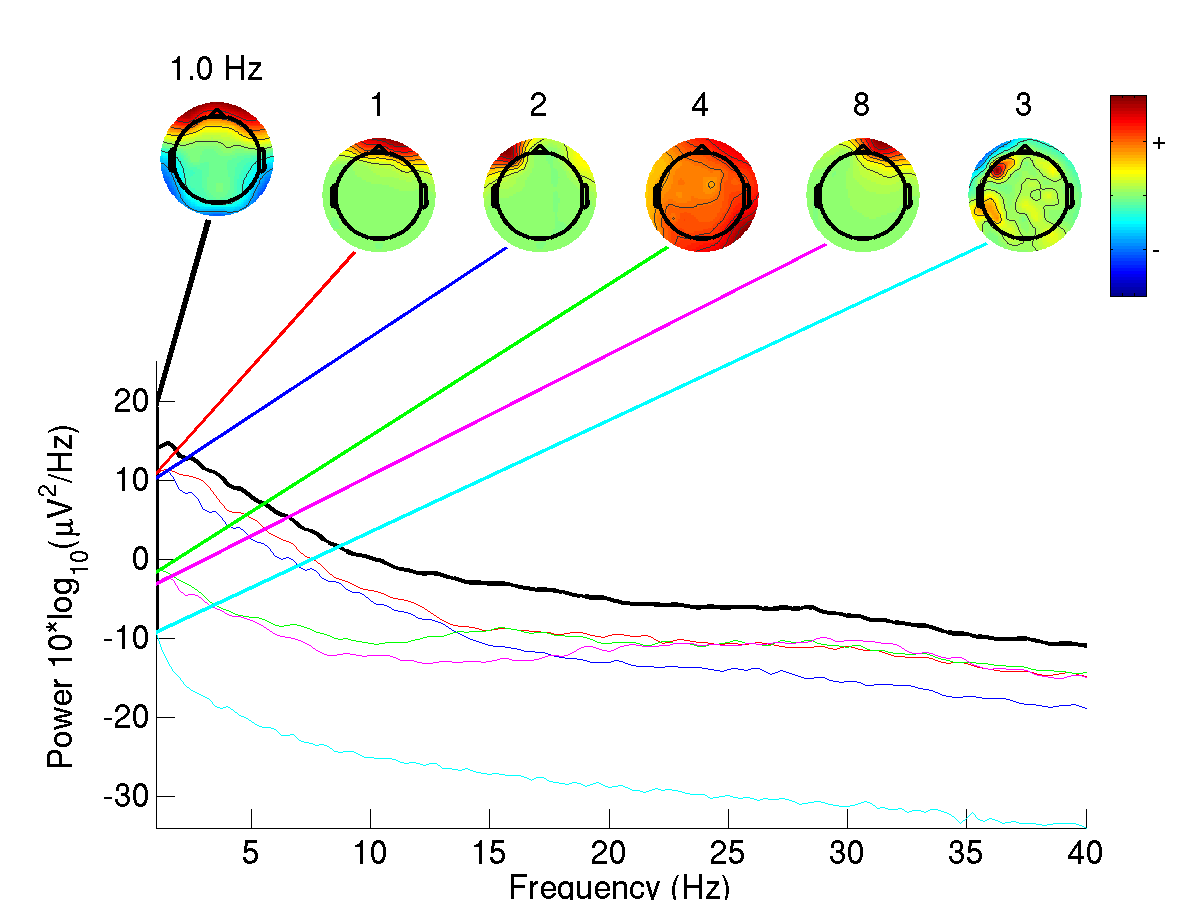
**

**Participant 4:** (left) Equivalent dipole locations of all excluded independent components. (right) Scalp map and power spectra of the first five excluded components.

**
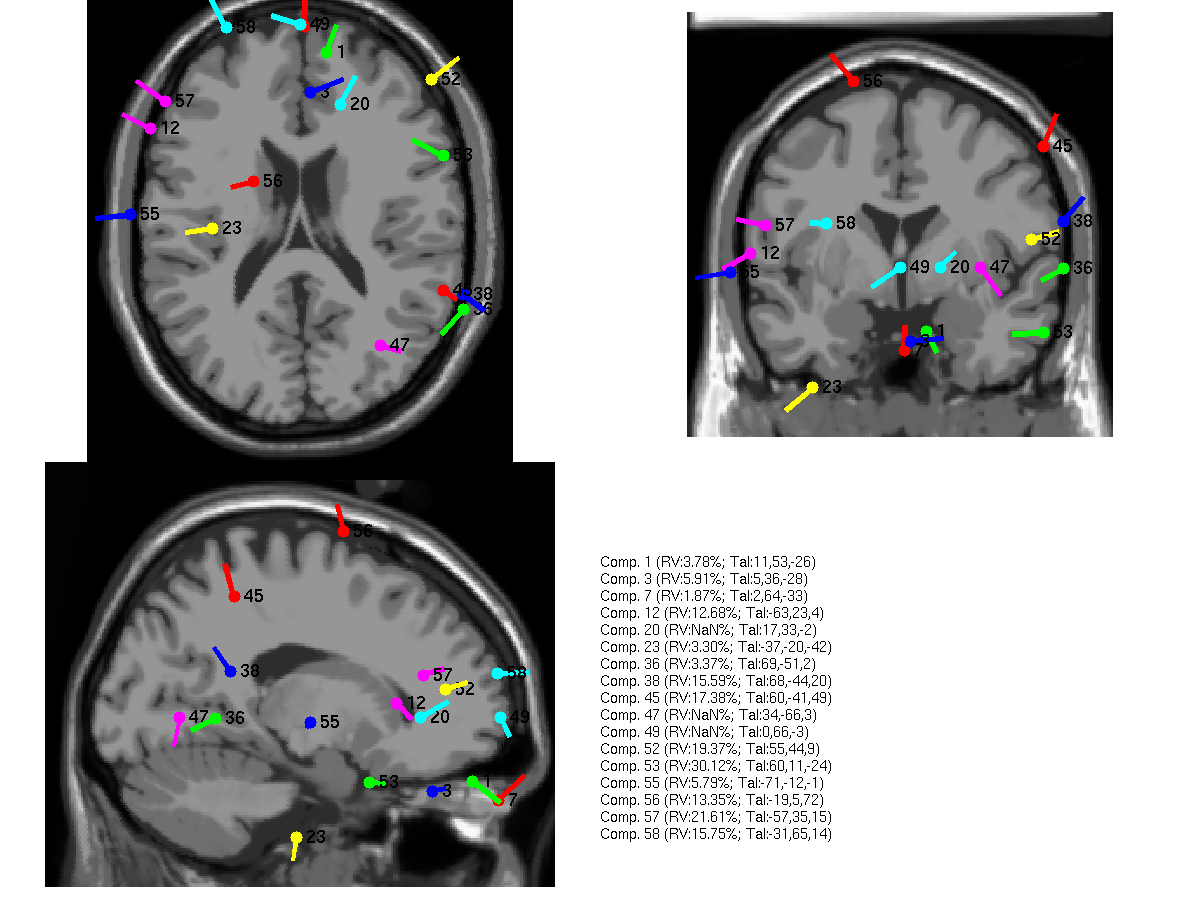
** **
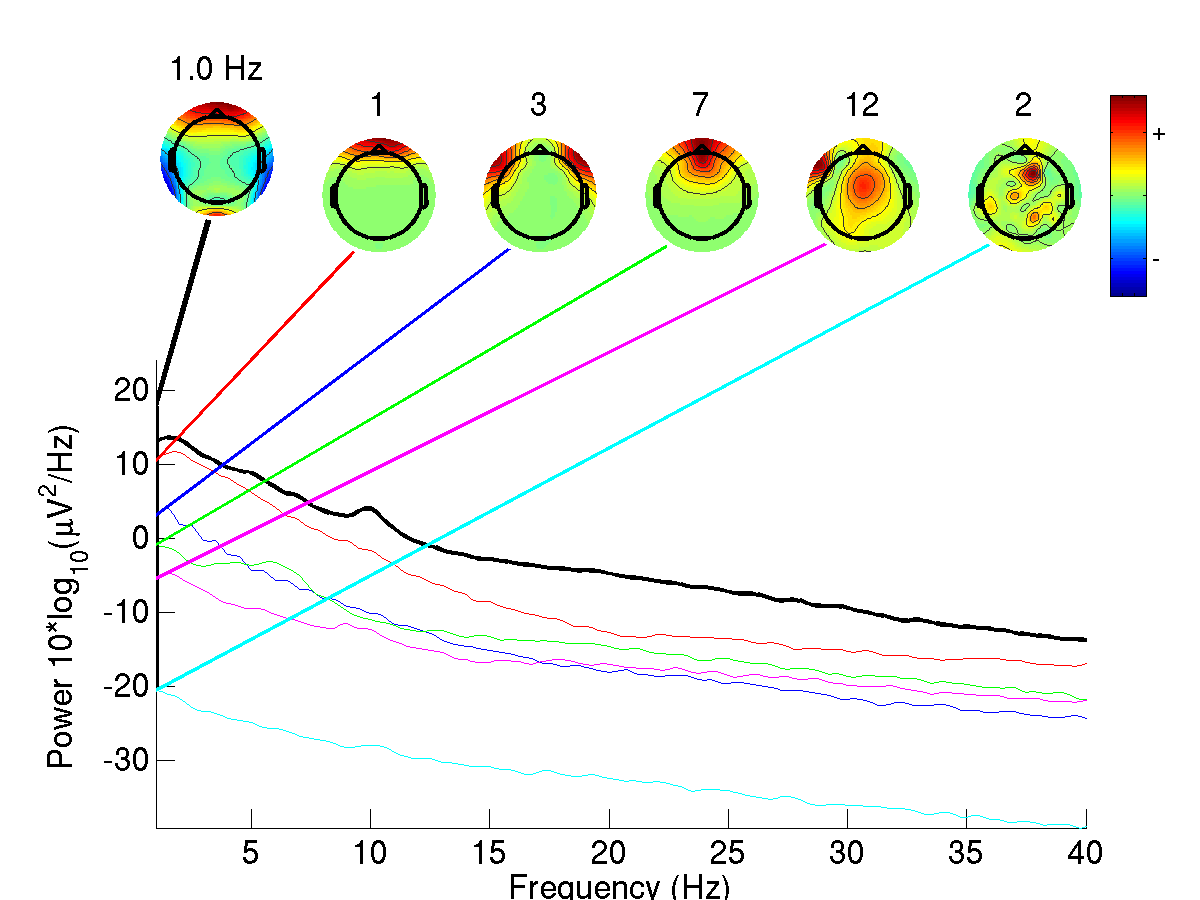
**

**Participant 5:** (left) Equivalent dipole locations of all excluded independent components. (right) Scalp map and power spectra of the first five excluded components.

**
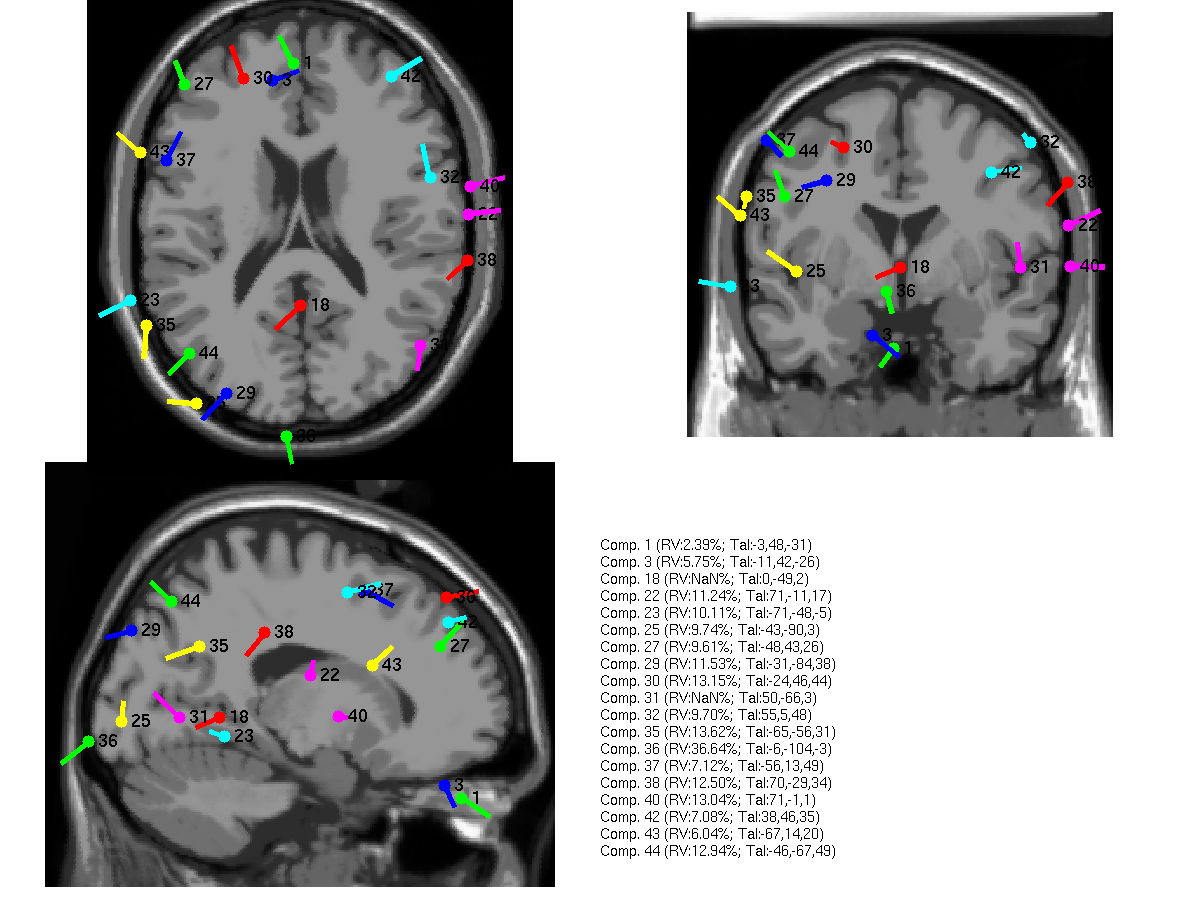

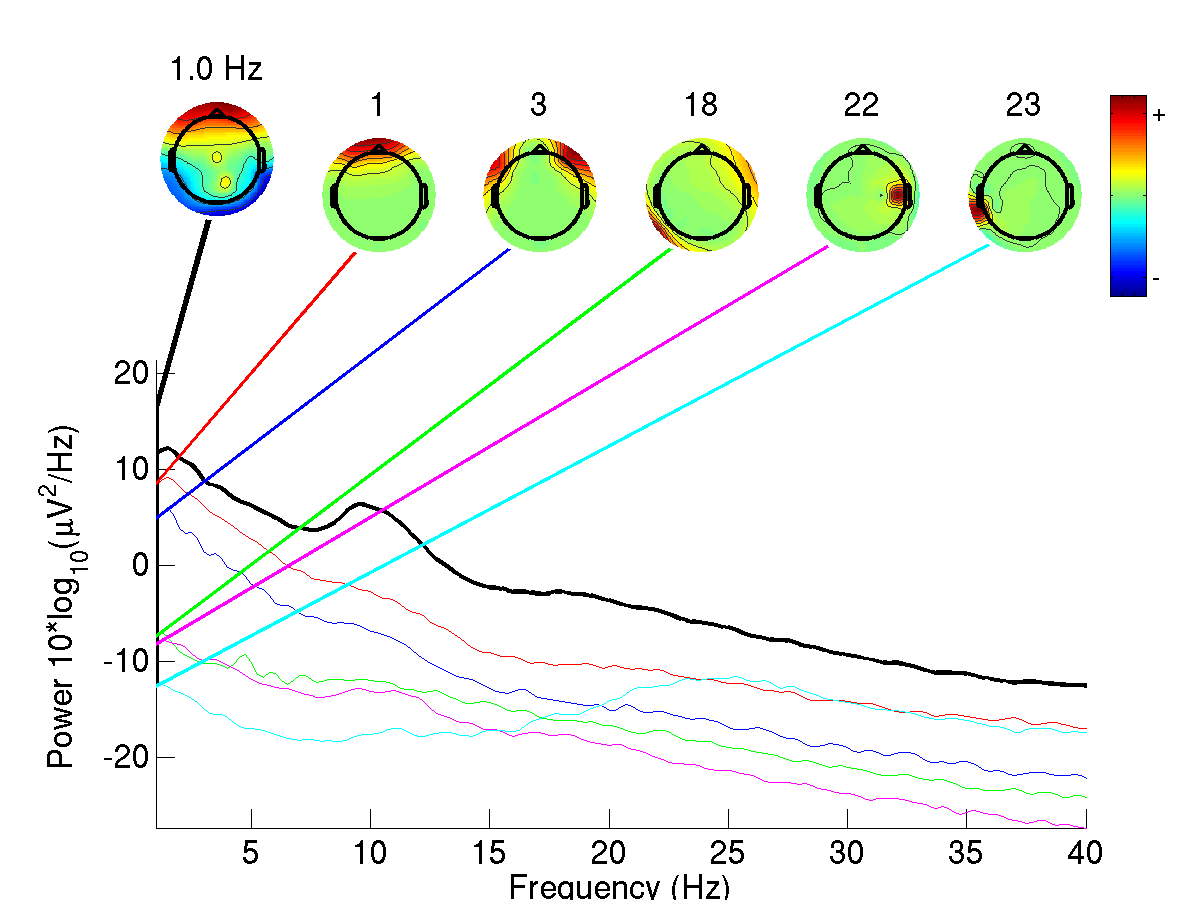
**

**Participant 3:** (left) Equivalent dipole locations of all excluded independent components. (right) Scalp map and power spectra of the first five excluded components.

**
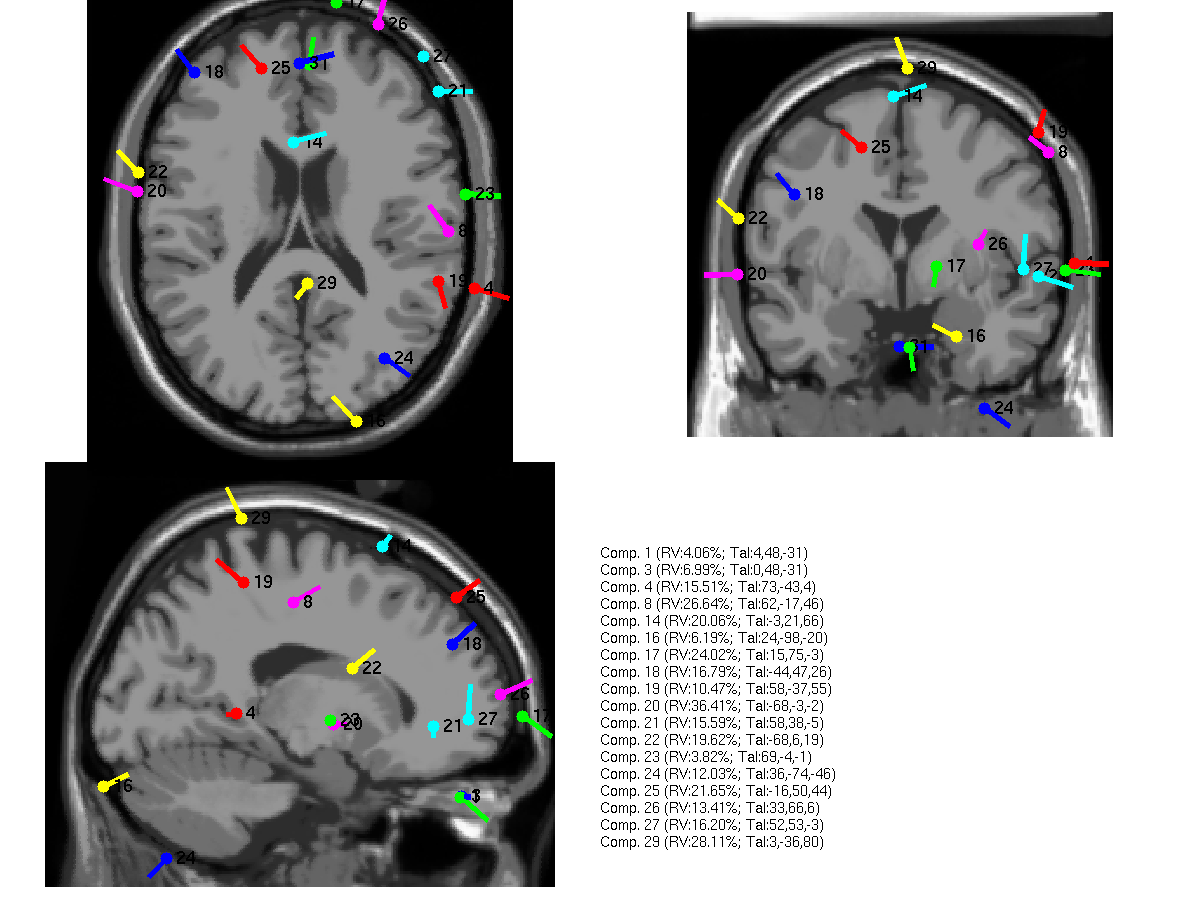

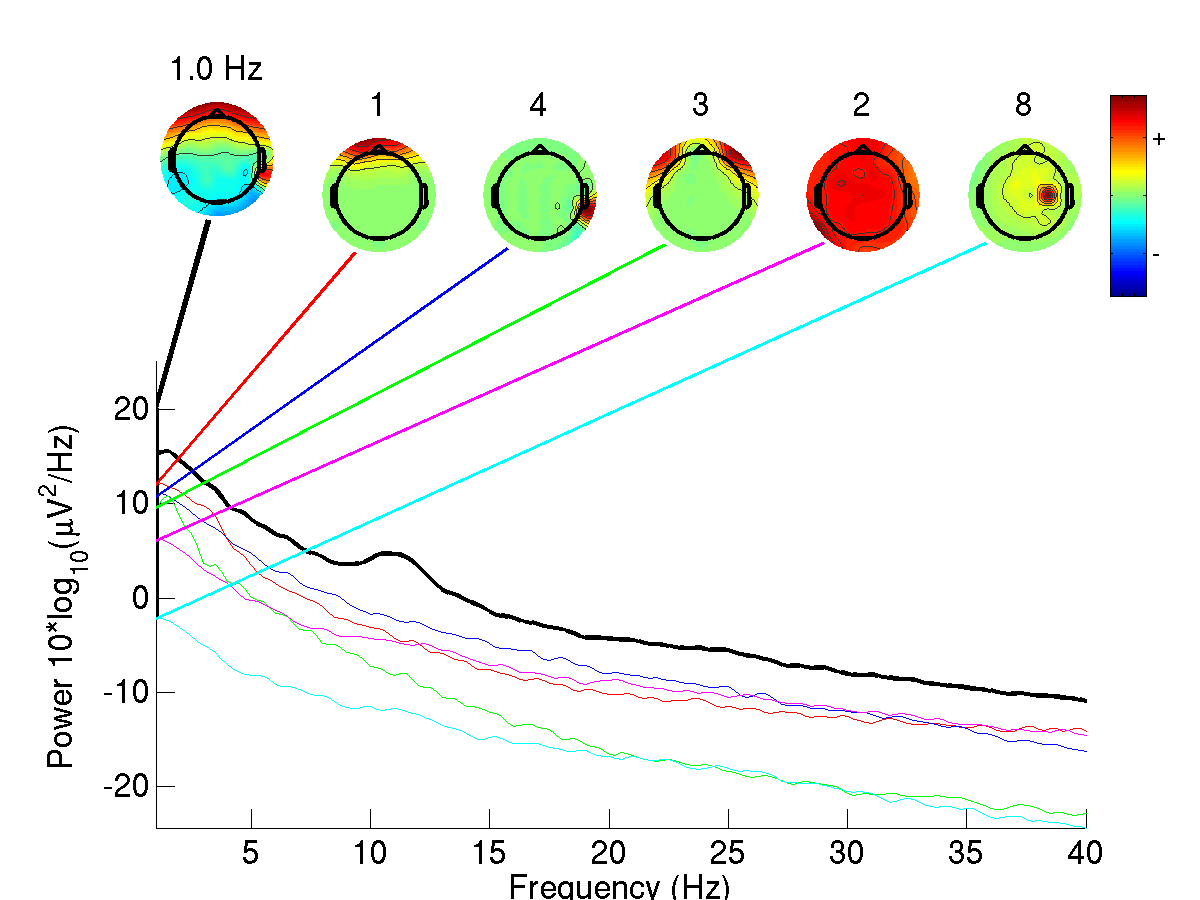
**

**Participant 7:** (left) Equivalent dipole locations of all excluded independent components. (right) Scalp map and power spectra of the first five excluded components.

**
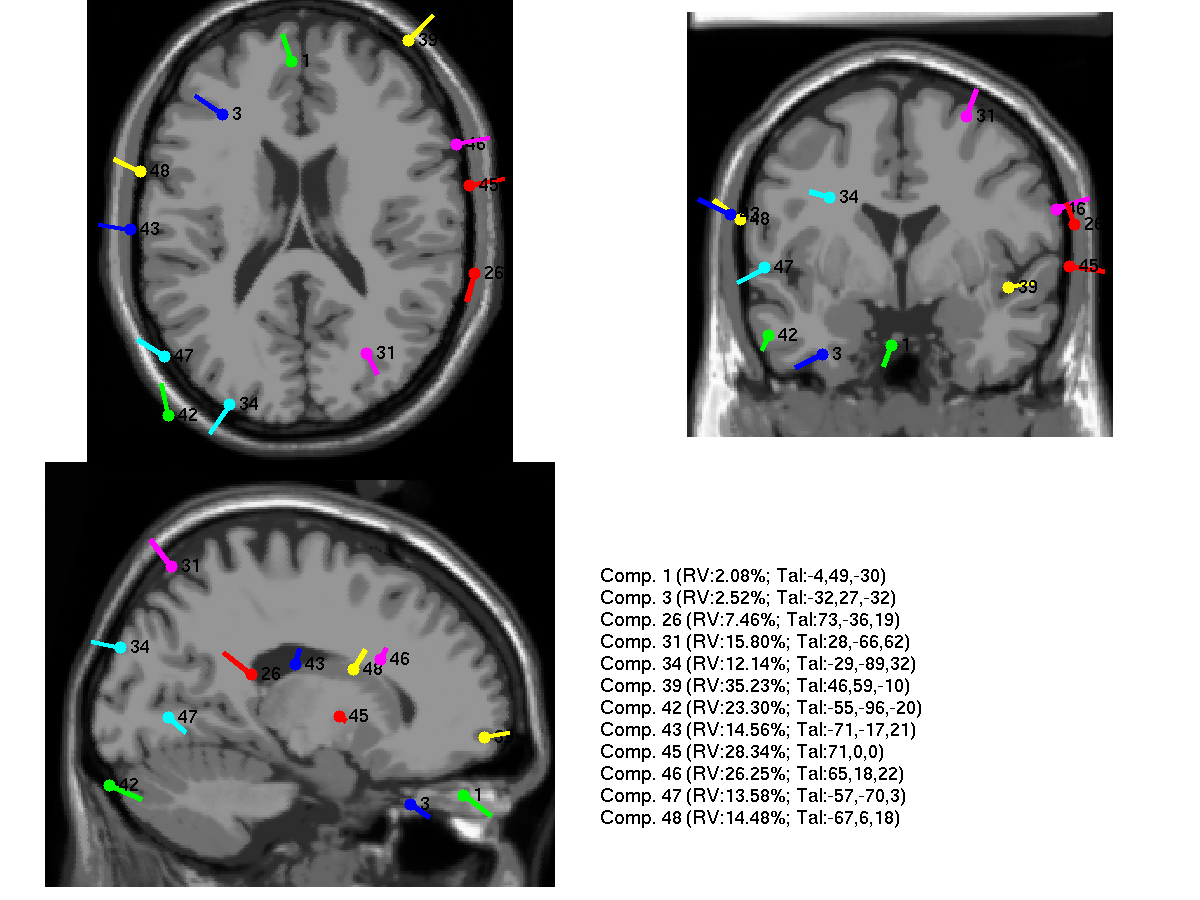

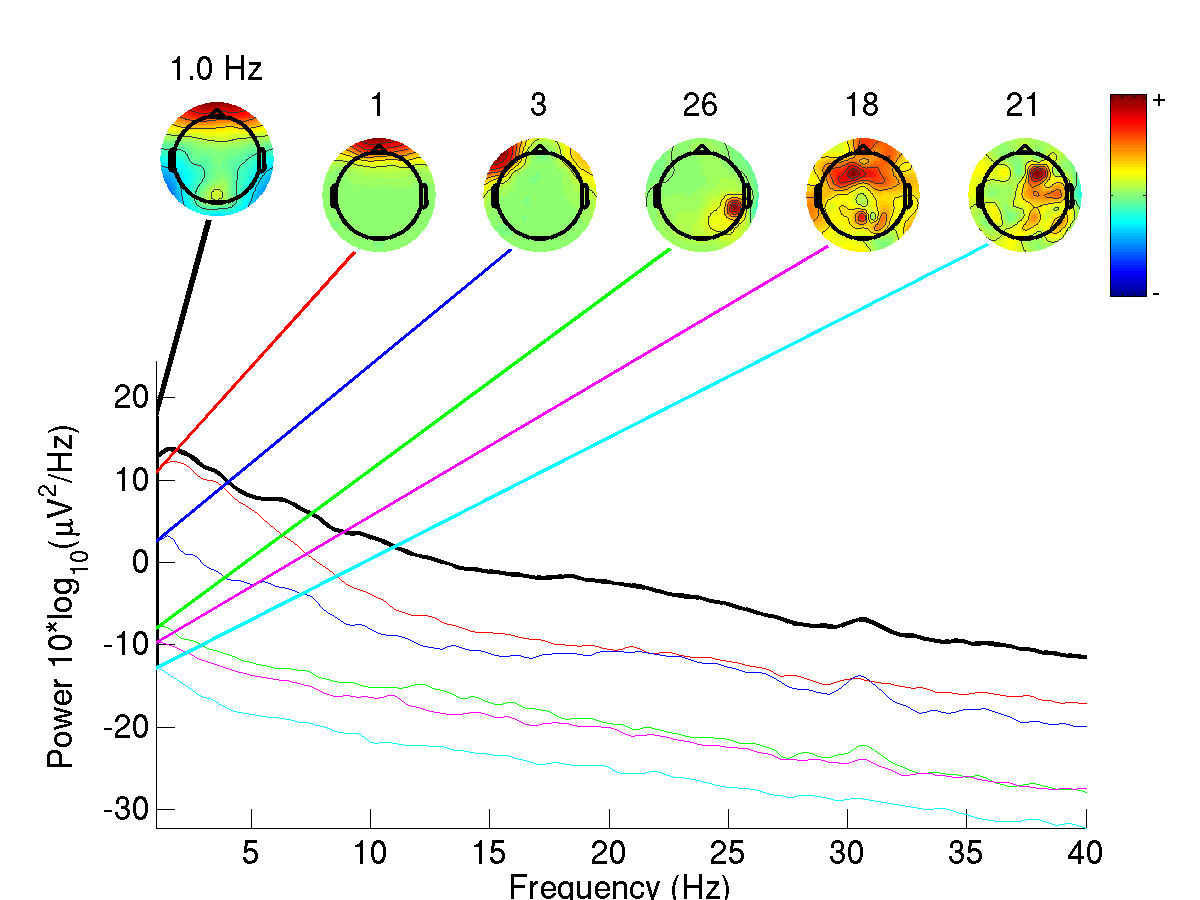
**

**Participant 8:** (left) Equivalent dipole locations of all excluded independent components. (right) Scalp map and power spectra of the first five excluded components.

**
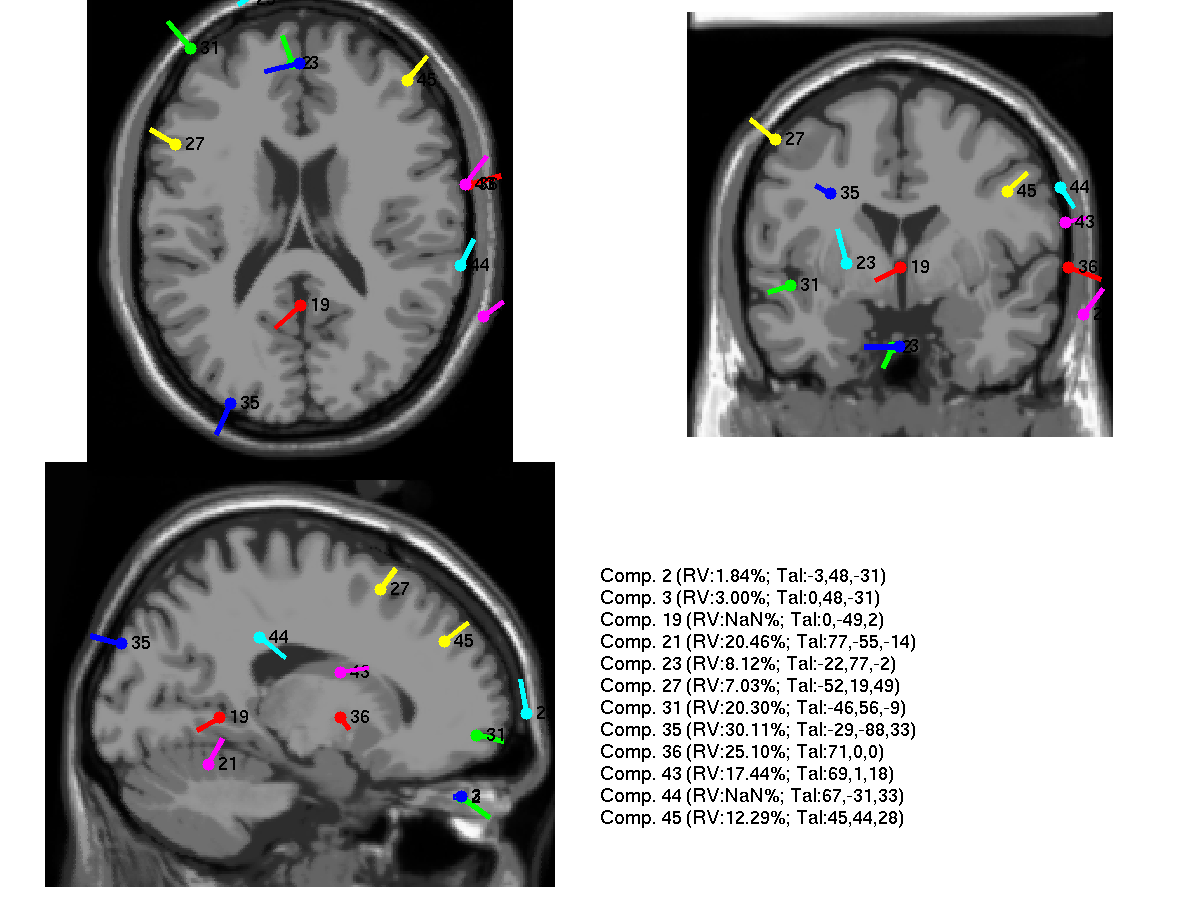

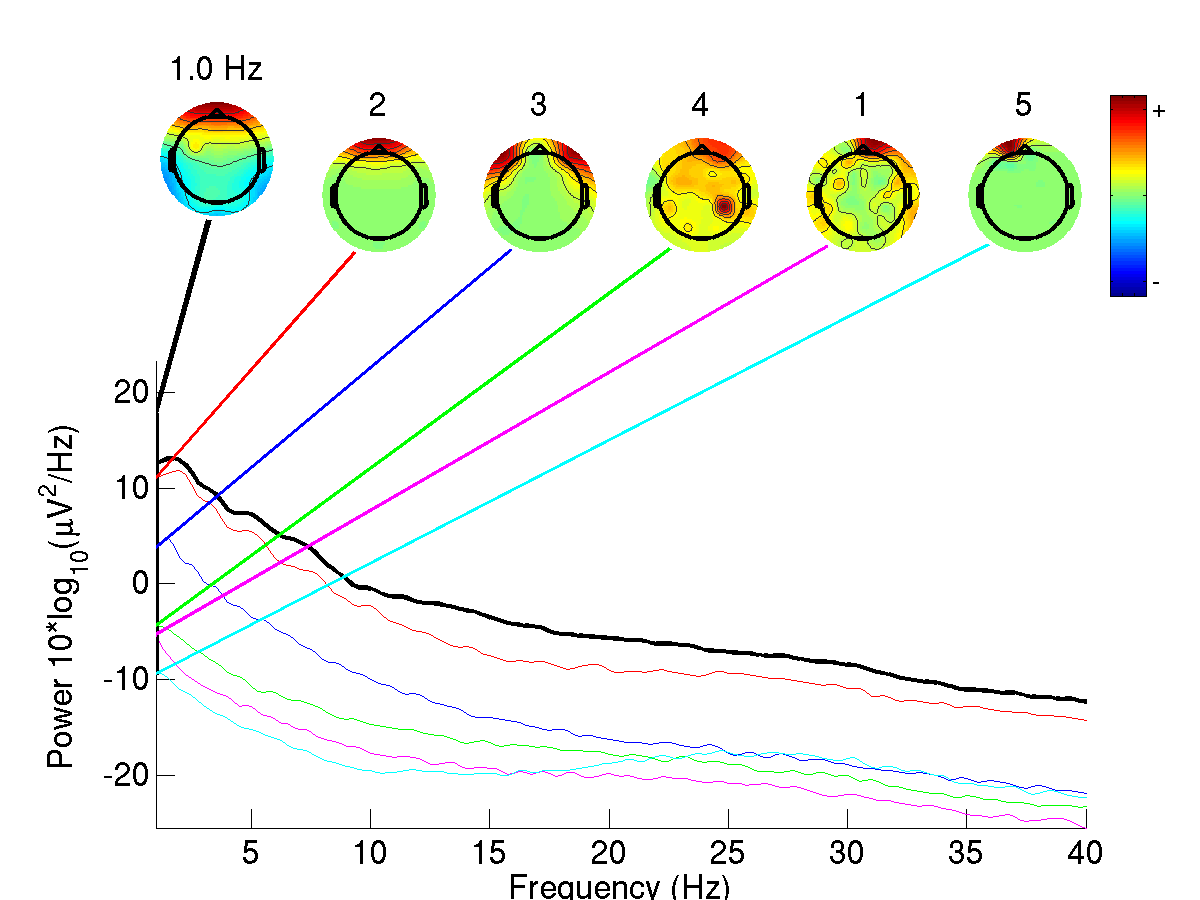
**

**Participant 6:** (left) Equivalent dipole locations of all excluded independent components. (right) Scalp map and power spectra of the first five excluded components.

**
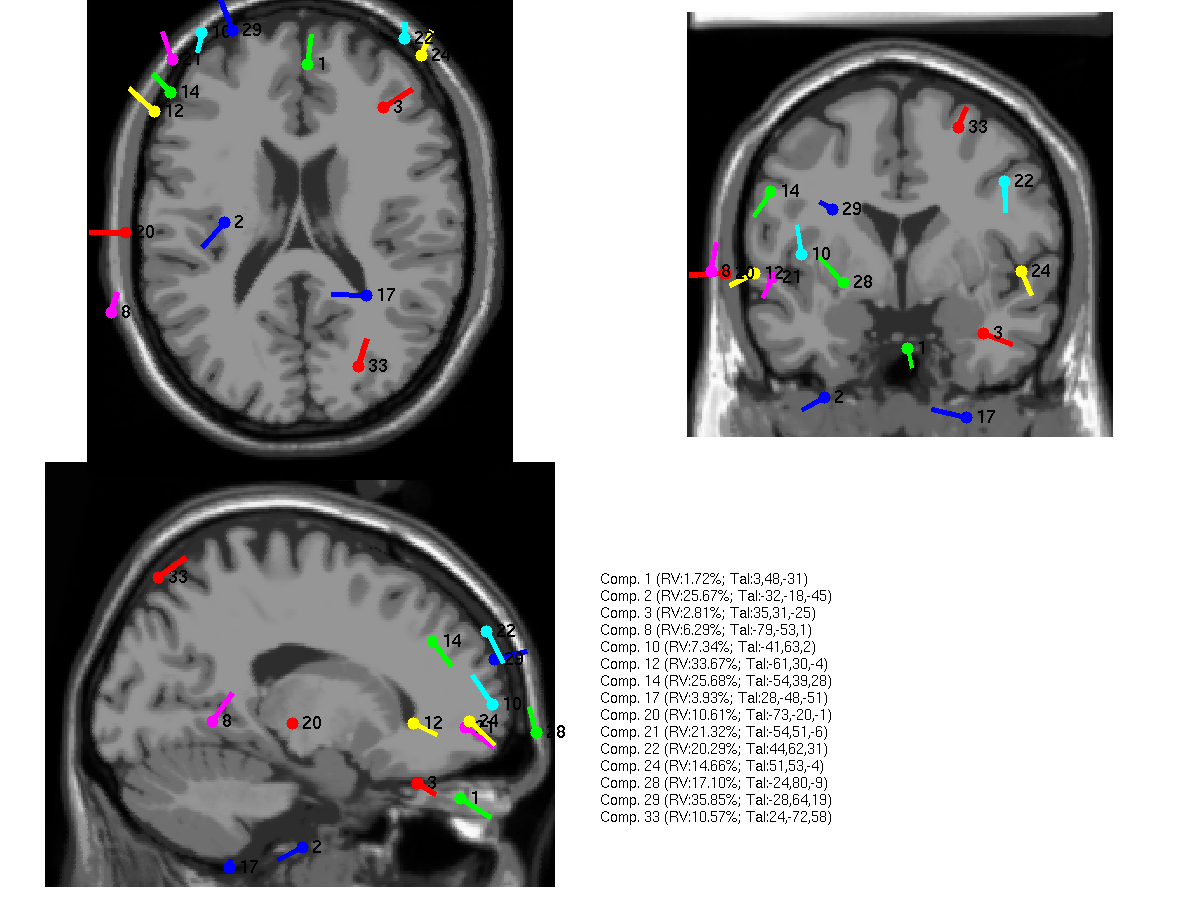

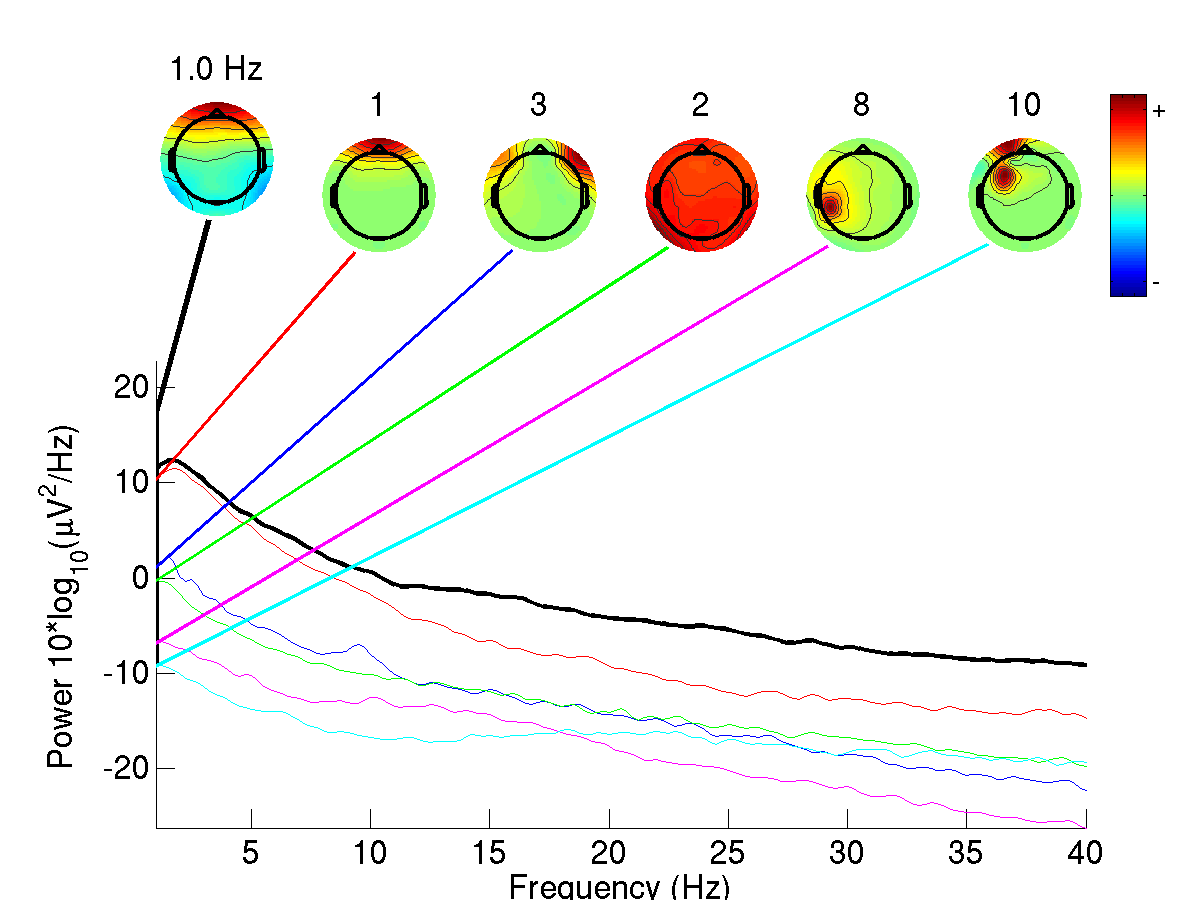
**

**Participant 10:** (left) Equivalent dipole locations of all excluded independent components. (right) Scalp map and power spectra of the first five excluded components.

**
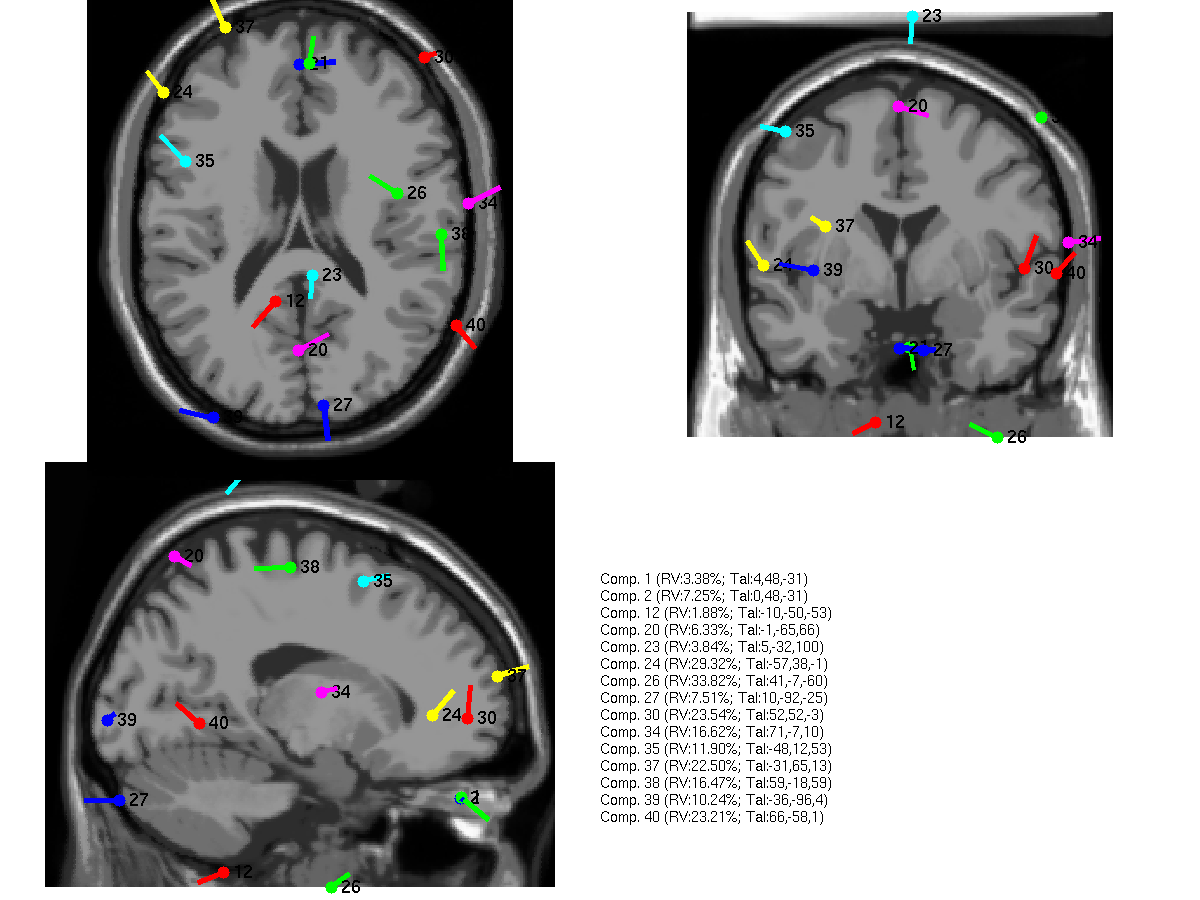

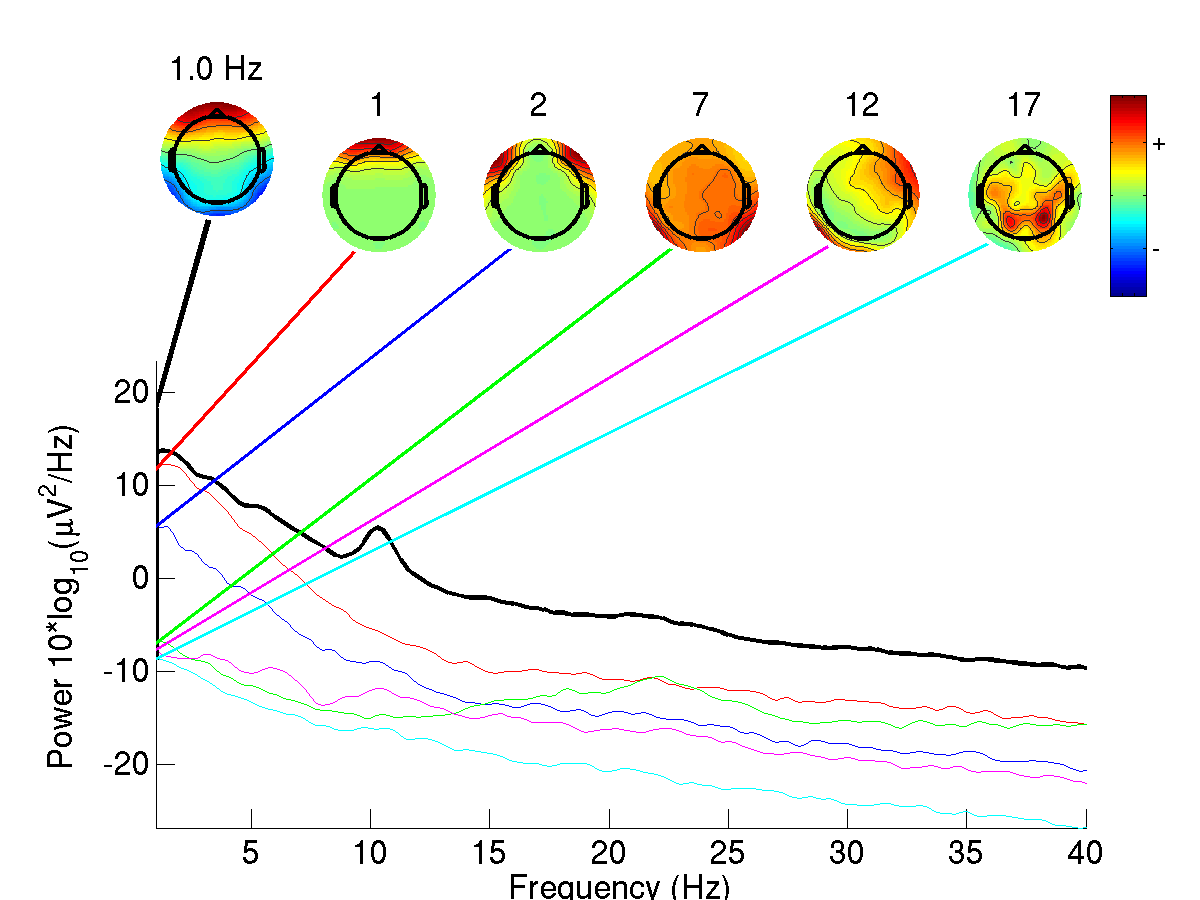
**

**Participant 11:** (left) Equivalent dipole locations of all excluded independent components. (right) Scalp map and power spectra of the first five excluded components.

**
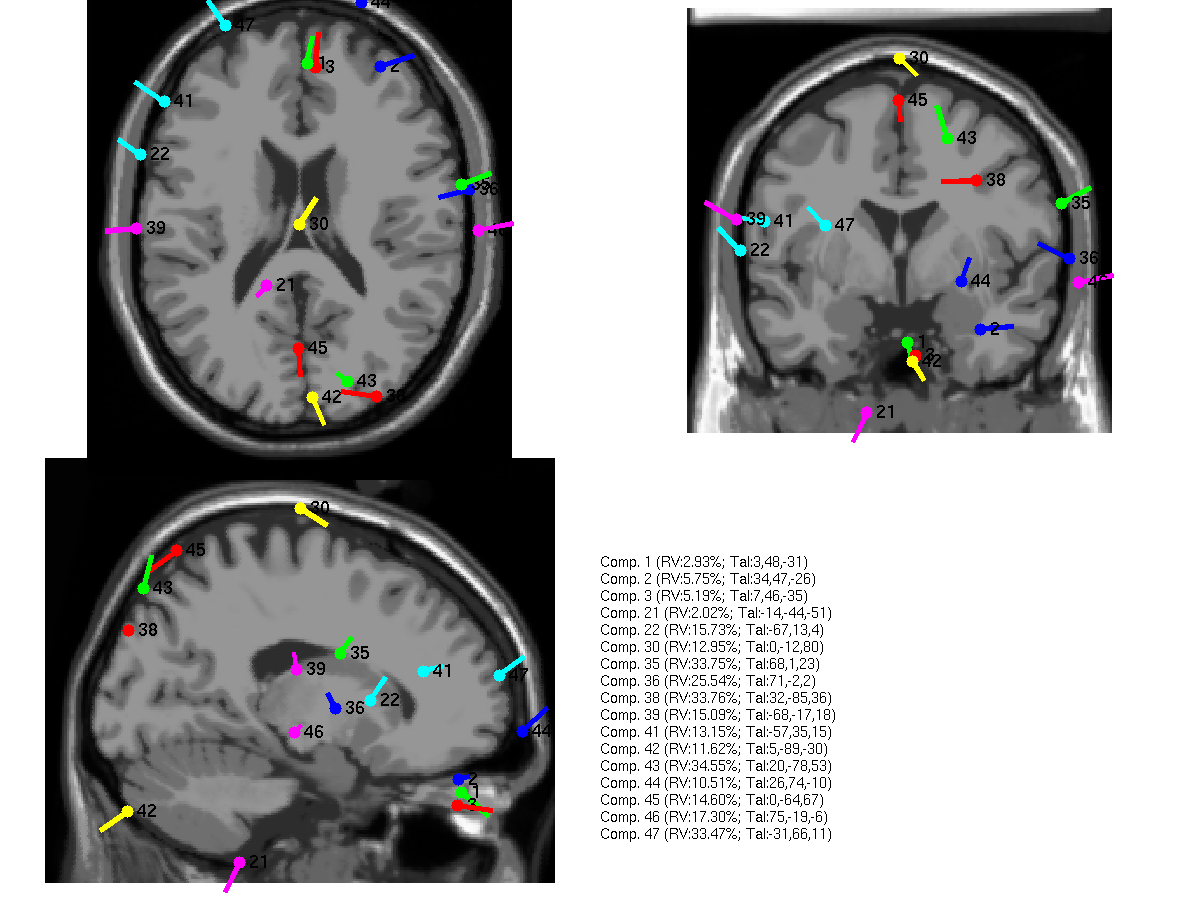

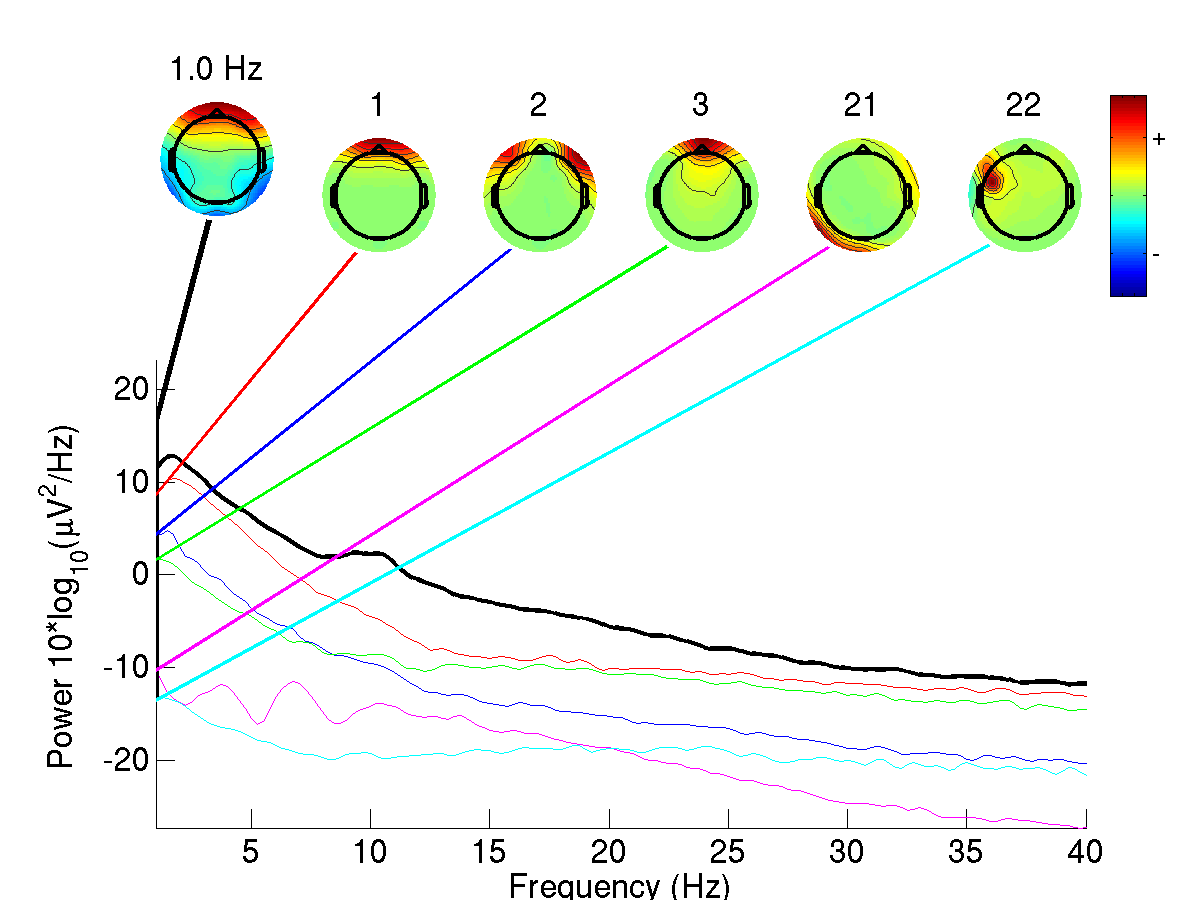
**

**Participant 9:** (left) Equivalent dipole locations of all excluded independent components. (right) Scalp map and power spectra of the first five excluded components.

**
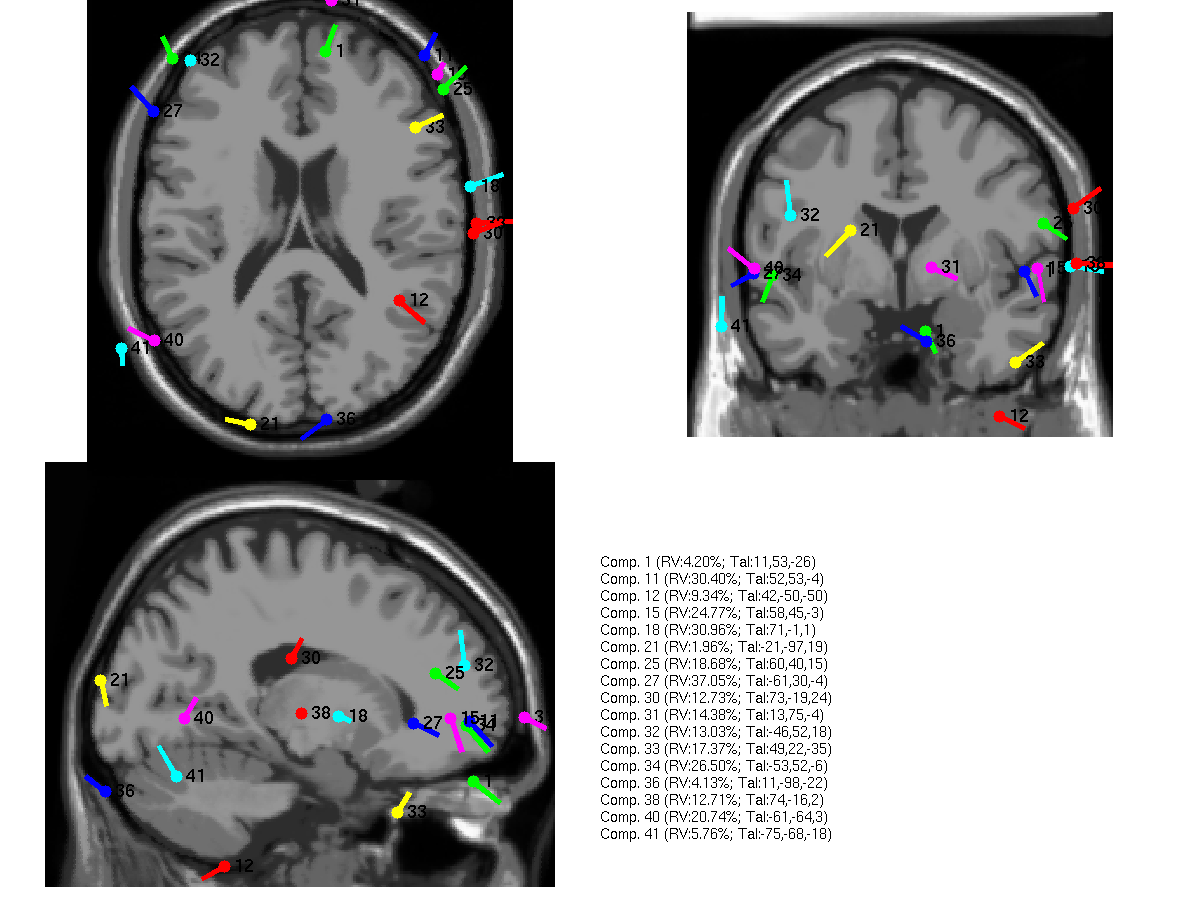

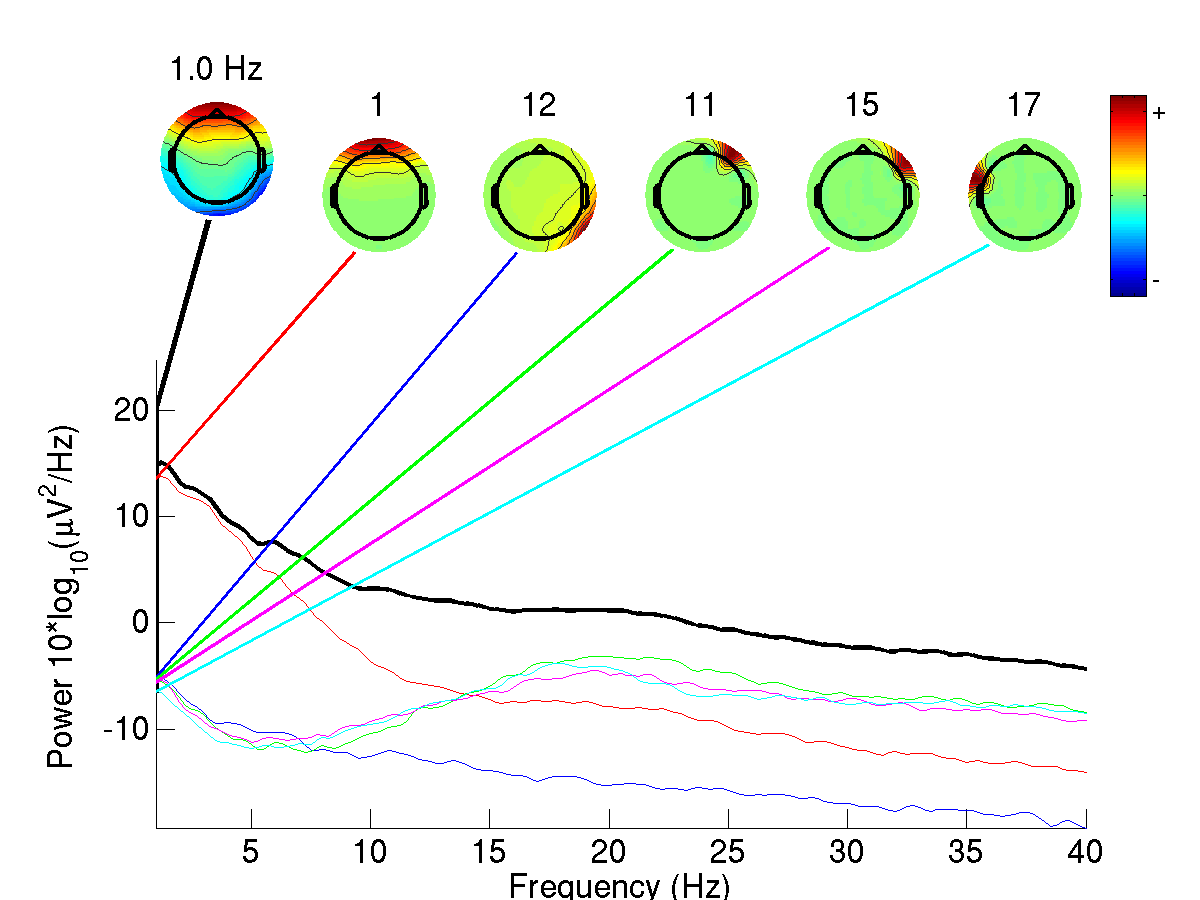
**

**Participant 13:** (left) Equivalent dipole locations of all excluded independent components. (right) Scalp map and power spectra of the first five excluded components.

**
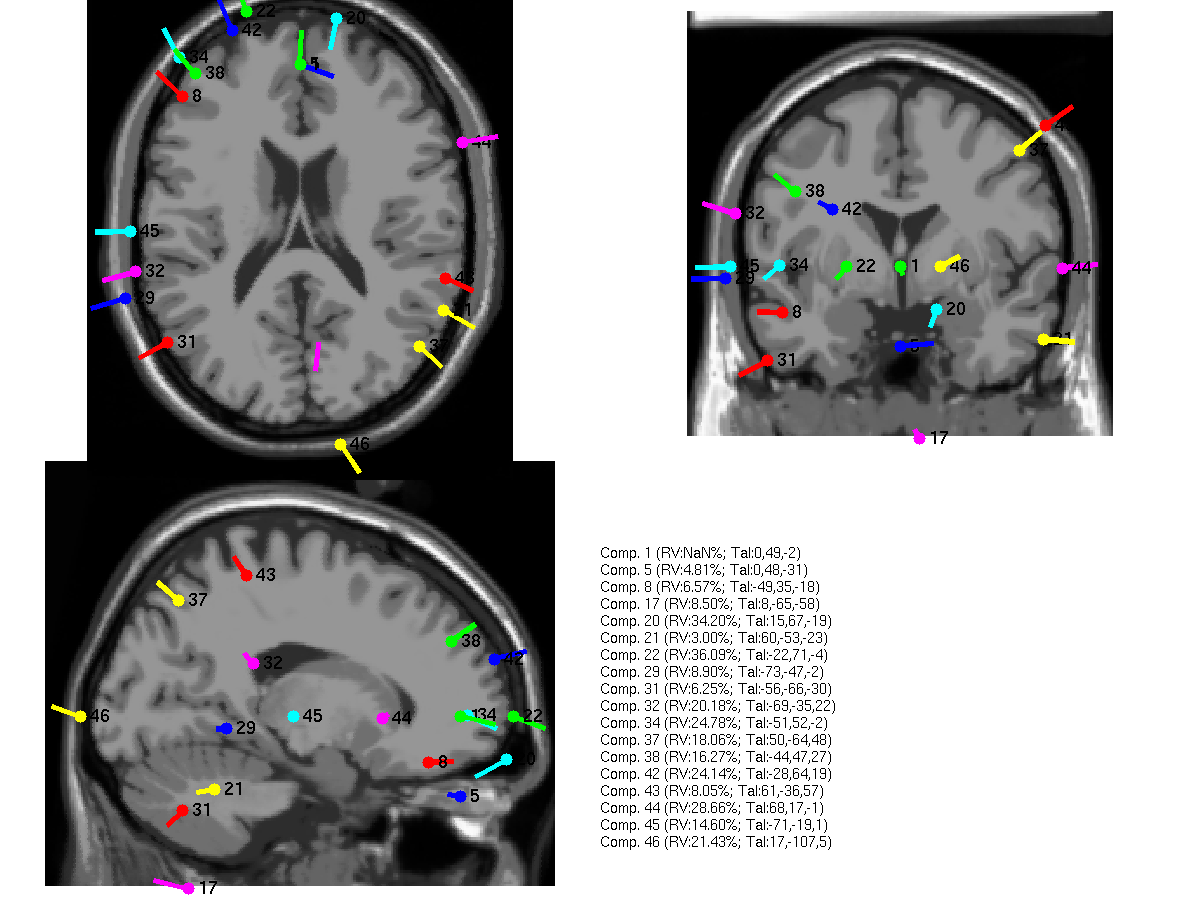

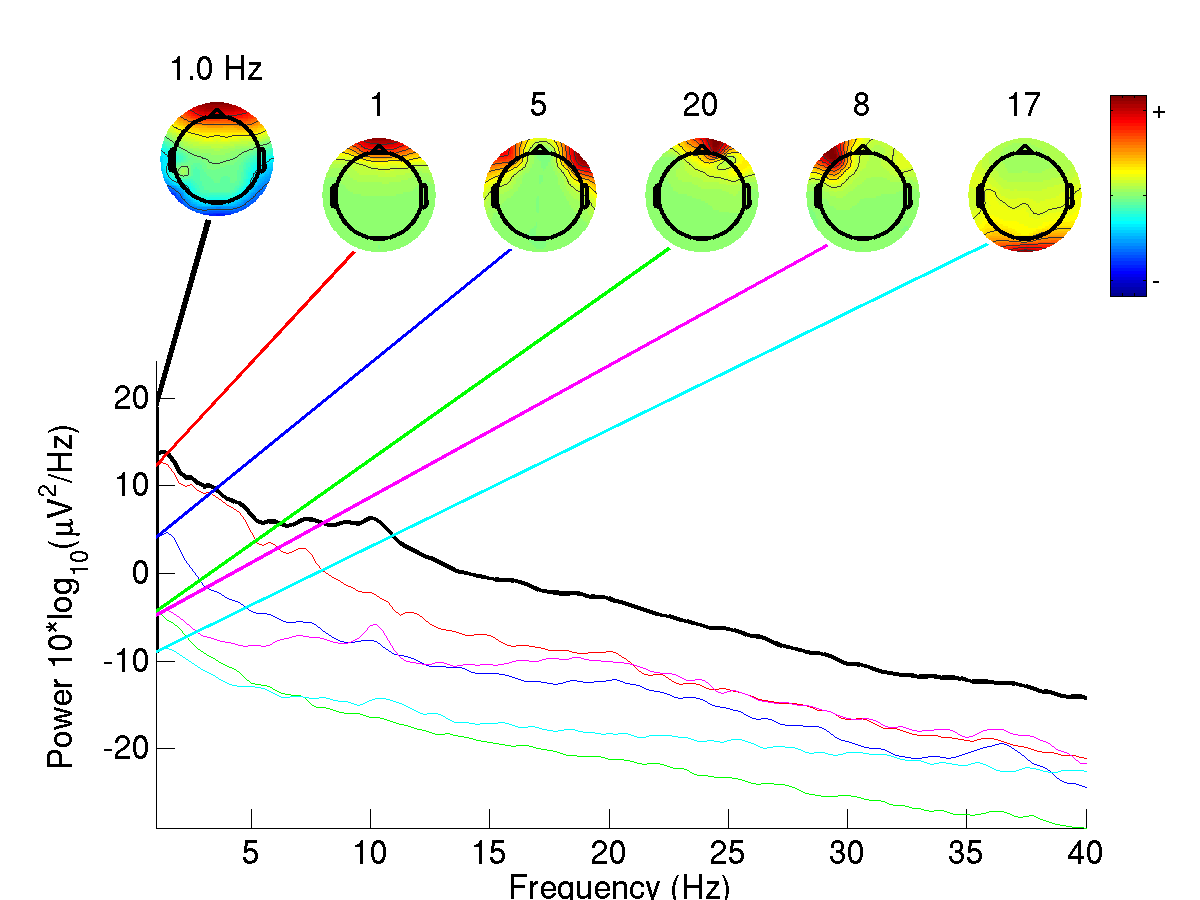
**

**Participant 14:** (left) Equivalent dipole locations of all excluded independent components. (right) Scalp map and power spectra of the first five excluded components.

**
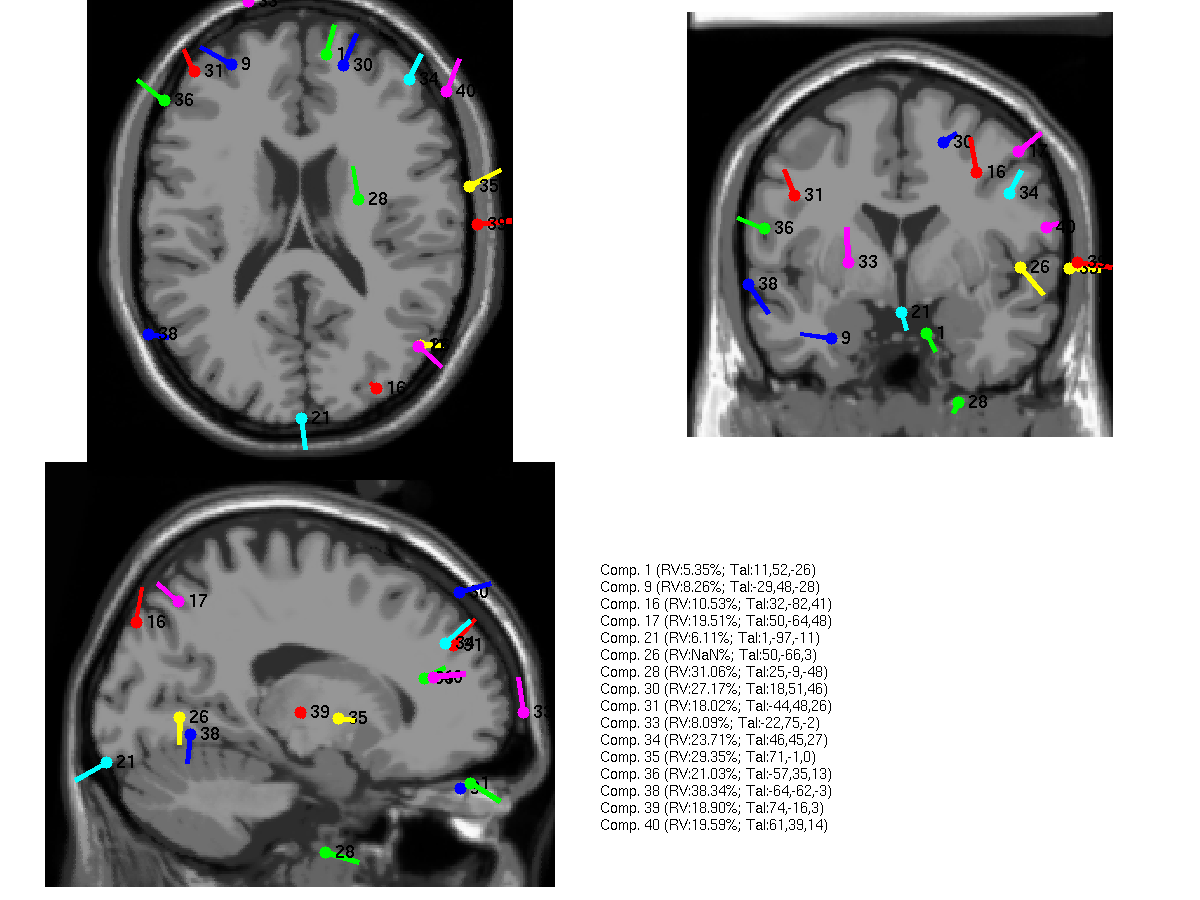

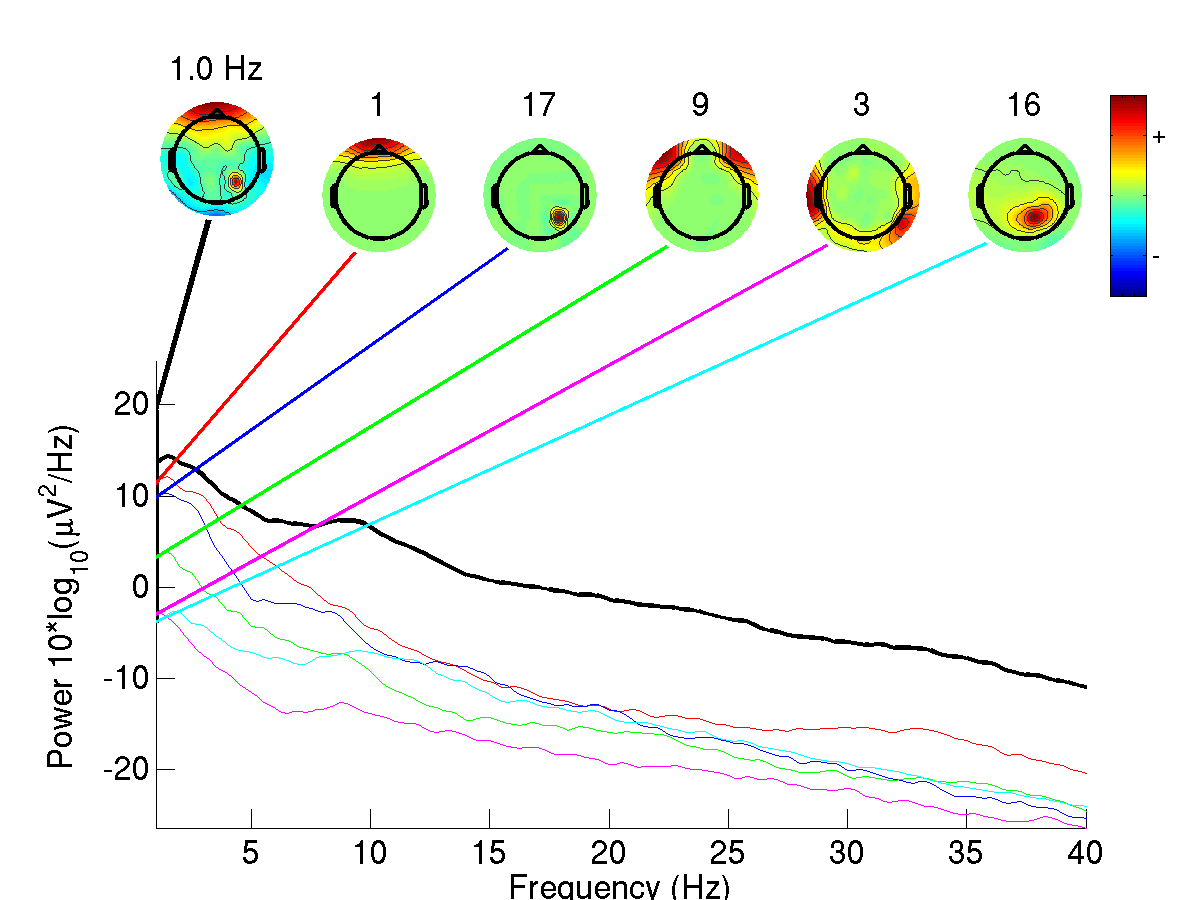
**

**Participant 12:** (left) Equivalent dipole locations of all excluded independent components. (right) Scalp map and power spectra of the first five excluded components.

1. * Corresponding Author: [jonathan.o.touryan.civ@mail.mil](mailto:jonathan.o.touryan.civ@mail.mil), (410) 278-4329 [↑](#footnote-ref-1)
